# Supplementary material for: High-frequency vs. low-frequency MIDI-assisted group music therapy in psychiatric inpatients: A randomized controlled trial
Source: PLoS One. 2026 Apr 17;21(4):e0317950. doi: 10.1371/journal.pone.0317950 (PMC13089878; doi:10.1371/journal.pone.0317950)
Supplement: S1 Appendix — (DOCX) [file pone.0317950.s001.docx]

Multisensory Group Music Therapy with PLAYTRON for Psychiatric inPatients.

1. **Why (Rationale and Theory)**

The intervention was designed to promote emotional regulation, sustained attention, creativity, and group cohesion among psychiatric inpatients. It was grounded in the principles of active music therapy, integrating progressive muscle relaxation techniques and multisensory stimulation to support therapeutic engagement. The approach aimed to explore novel pathways for connecting with the environment through sensory and creative experiences, with the goal of reducing catastrophic thinking and enhancing participants’ capacity for awe and presence in the moment.

1. **What (Materials)**

The materials used in the intervention included a Playtron MIDI controller connected to conductive objects, such as fruits, water, and plants, which were assigned specific sounds via a mobile application. Traditional musical instruments, including a portable piano and guitar, were also incorporated to facilitate musical interaction. The intervention took place in an indoor therapeutic space designated for occupational therapy, designed to provide a controlled and conducive environment for the activities. Additionally, relaxation materials, such as a quiet setting and guided instructions, were used to support the relaxation and emotional synchronization phases of the therapy.

1. **What (Procedures)**

The music therapy sessions followed a structured and progressive format that integrated musical interaction with relaxation and emotional synchronization techniques. Each session lasted 30 minutes and was divided into four sequential phases: warm-up, group synchronization, main activity, and closure.

The **warm-up phase** aimed to foster mutual recognition among participants and establish group cohesion. A standardized welcome song was used to create a safe and welcoming environment. The repetition of the same musical piece across sessions allowed participants to associate the melody with emotional safety and familiarity, thereby facilitating attentional focus and presence in the “here and now.”

The **second phase** involved the practice of Progressive Muscle Relaxation (PMR), wherein participants synchronized breathing with muscle activation and relaxation. Using guided thoracic breathing, individuals were encouraged to perform a bodily "scan" to identify areas of tension and apply intentional relaxation. This practice served to prepare participants both physically and emotionally for the creative components of the session by promoting internal regulation.

The **main activity** introduced the PLAYTRON, a MIDI controller that enables interaction with conductive objects—such as fruits—transformed into musical instruments. This multisensory engagement promoted sustained attention, group role-play, creativity, and sensory exploration. The use of everyday items as musical devices added novelty and playfulness to the therapeutic setting.

During the **closing phase**, a farewell song was performed collaboratively, integrating participants' voices with the sounds produced via the PLAYTRON. This ritualistic closure reinforced the group process, accompanied by multisensory stimuli such as the scent of fruits and plants, and concluded with the optional tasting of the fruit-instrument. This final element supported emotional integration, strengthened group cohesion, and provided a positive affective resolution to the session.

1. **Who Provided**

The intervention was directed by a professional with a master’s degree in music therapy from the University of La Rioja, validated by the Colombian Ministry of Education. The music therapist was accompanied by a physician trained in psychological first aid, as well as by the principal invmidiestigator—a psychiatry resident—who was qualified to intervene in the event of any psychological destabilization or clinical need during the sessions.

1. **How (Mode of Delivery)**

The intervention was delivered through group-based, in-person sessions held in a controlled therapeutic environment. Each session accommodated a maximum of 16 participants per group for the creation of musical compositions in ensembles through the use of conductive objects, aiming to engage them creatively and sensorially. Traditional musical instruments such as piano and guitar were used alongside a Playtron MIDI controller, which was connected to conductive objects like fruits, water, and plants. Each object was assigned a unique sound via a mobile application, encouraging collaborative music-making and enhancing both individual creativity and group expression. The multisensory setup fostered a playful, exploratory experience, and the sessions culminated in a ritualized closure, where participants ate the fruit used during the session, reinforcing a sense of ownership and connection to the musical process.

The application of MIDI-enabled devices in music therapy has been documented in first world countries; but to the best of our Knowledge this is the first documented intervention involving psychiatric patients in which a MIDI controller connected to fruits or other conductive materials was employed to enhance environmental interaction and stimulate a sense of wonder. This innovative approach represents a great contribution both in the Colombian context and globally.

1. **Where (Location)**

The intervention was conducted in a designated indoor space of the Montserrat Clinic, specifically allocated for occupational therapy sessions with psychiatric inpatients. The environment was structured to provide a safe, controlled, and therapeutically appropriate setting for group-based multisensory music therapy.

1. **When and How Much (Duration, Frequency)**

Patients were recruited consecutively between August 1st and September 4th upon admission to the psychiatric unit. Each session lasted 30 minutes. For the high-intensity group, sessions were conducted once per day for five consecutive days, while the low-intensity group participated in one session per day. At the end of the intervention, the patients who belonged to the low intensity group and who continued in the hospitalization process could attend music therapy to complete a total of 5 sessions, thus complying with an equitable treatment of all the participants of the experiment.

1. **Tailoring (Adaptations)**

The intervention protocol was standardized across all participant groups, and no individual adaptations were made to the core structure or delivery of the sessions. Although the therapeutic team closely monitored each participant's engagement, sensory response, and emotional state throughout the sessions, these observations did not lead to modifications in the intervention design. This consistent approach ensured fidelity to the therapeutic model and documentation of individual progress.

1. **Modifications (Changes during the Study)**

No modifications to the structure, content, or delivery of the intervention were made during the course of the study. The implementation followed the originally designed protocol without deviation.

1. **How Well - Planned (Fidelity Planned)**

Intervention fidelity was ensured through the use of a standardized session structure consisting of four clearly defined phases: warm-up, group synchronization, main activity, and closure. Consistency across sessions was maintained through continuous supervision by a multidisciplinary team, including the music therapist, the principal investigator (a psychiatry resident), and a physician. This team-based oversight aimed to guarantee adherence to the protocol and uniformity in the delivery of the intervention across all participant groups.

1. **How Well - Actual (Fidelity Actual)**

No formal fidelity assessment was conducted during the implementation of the intervention. However, adherence to the planned session structure was informally monitored through attendance records, direct supervision by the clinical and research team, and consistent observation of session flow. While these measures supported procedural consistency, a structured fidelity evaluation tool was not employed.
