# Supplementary material for: High-frequency vs. low-frequency MIDI-assisted group music therapy in psychiatric inpatients: A randomized controlled trial
Source: PLoS One. 2026 Apr 17;21(4):e0317950. doi: 10.1371/journal.pone.0317950 (PMC13089878; doi:10.1371/journal.pone.0317950)
Supplement: S2 Appendix — (DOCX) [file pone.0317950.s002.docx]

**Appendix S2. Model diagnostics for linear mixed-effects models**

Model diagnostics were systematically evaluated for all linear mixed-effects models fitted for the depression,Appendix_S2_Model anxiety, and stress subscales of the DASS-21, separately for the high-intensity (Group 1) and low-intensity (Group 2) intervention groups. Across all six models, inspection of standardized residual QQ-plots showed an approximately normal distribution, with only minor deviations at the distribution tails and no extreme standardized residuals (|z| > 3). Residuals-versus-fitted plots revealed a random and symmetric dispersion of residuals around zero, with no evidence of systematic patterns or funnel-shaped structures, supporting the assumption of homoscedasticity. In addition, influence diagnostics indicated the absence of influential observations, as confirmed by Cook’s distance values and the lack of residuals exceeding conventional thresholds. Taken together, these graphical and numerical diagnostics support the adequacy and robustness of the linear mixed-effects modeling approach used for all outcomes and intervention groups.

Table S1 summarizes the standardized residual diagnostics for all linear mixed-effects models fitted for depression, anxiety, and stress outcomes, stratified by intervention group. Across all six models, the maximum absolute standardized residual ranged between 2.19 and 2.97, remaining below the conventional threshold of |3| used to flag extreme outliers. Importantly, no observations exceeded this threshold in any model, indicating the absence of extreme residuals. These results provide quantitative support for the graphical diagnostics, suggesting that none of the fitted models were unduly influenced by outlying observations and that the assumptions underlying the linear mixed-effects models were adequately met.

**Table S1.** Standardized residual diagnostics for linear mixed-effects models

| **Model** | **Max. absolute standardized residual** | **No. of \|z\| > 3** |
| --- | --- | --- |
| Depression_G1 | 2.19 | 0 |
| Depression_G2 | 2.71 | 0 |
| Stress_G1 | 2.45 | 0 |
| Stress_G2 | 2.23 | 0 |
| Anxiety_G1 | 2.97 | 0 |
| Anxiety_G2 | 2.34 | 0 |

Standardized residuals were examined to assess potential outliers. No model presented standardized residuals exceeding |3|, indicating the absence of extreme residuals.

**Figure S1.** QQ-plot of standardized residuals for the linear mixed-effects model of depression (DASS-21) in the high-intensity intervention group (Group 1).


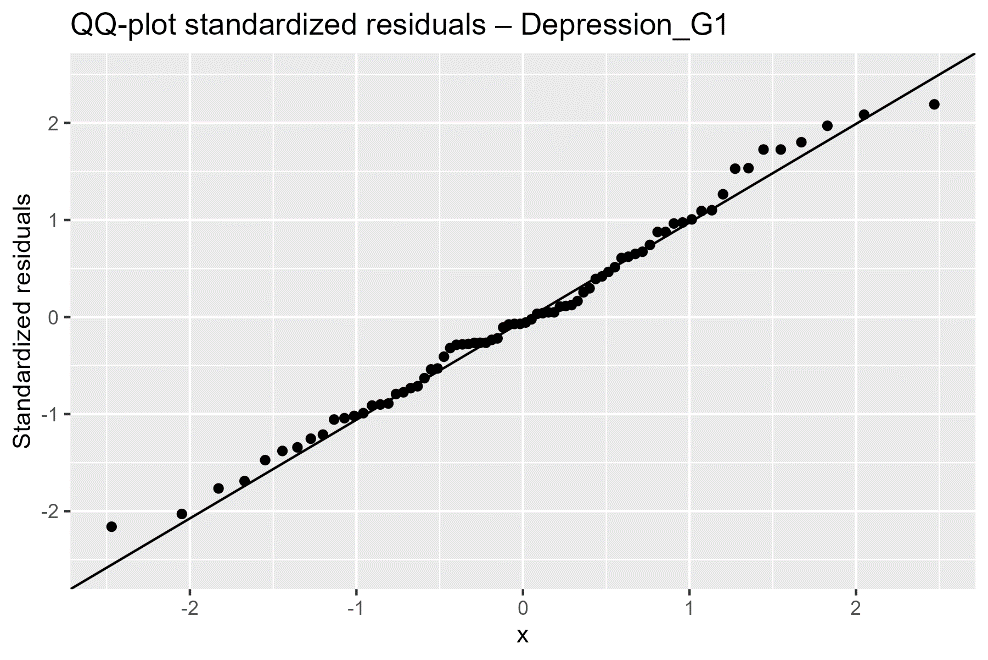


*The plot shows an approximately normal distribution of residuals, with no extreme deviations from the theoretical quantiles.*

**Figure S2.** Residuals versus fitted values for the linear mixed-effects model of depression (DASS-21) in the high-intensity intervention group (Group 1).


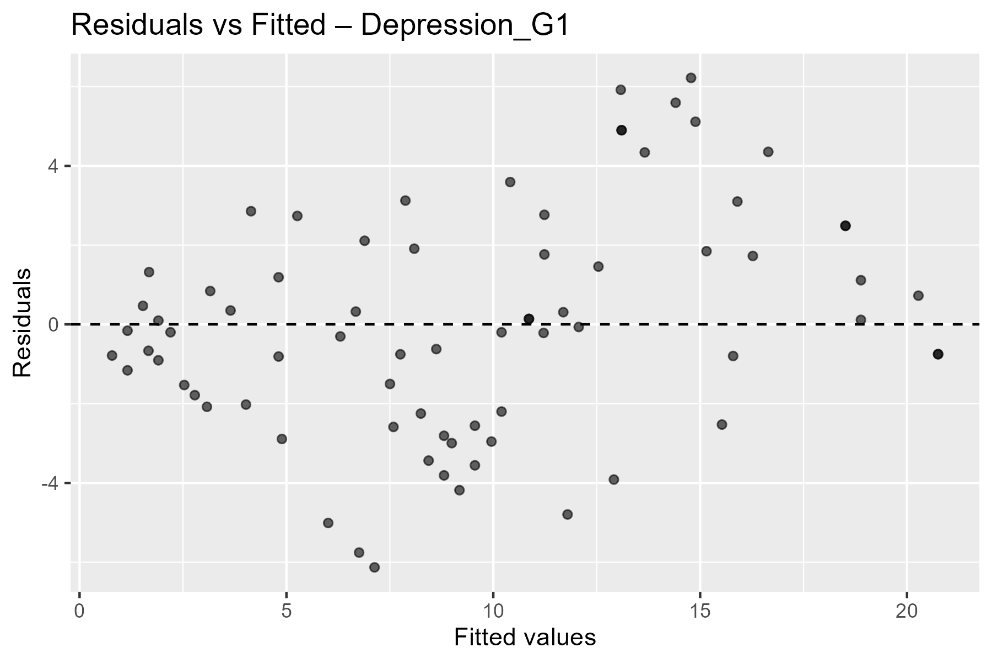


*Residuals are randomly distributed around zero, supporting the assumption of homoscedasticity.*

**Figure S3.** QQ-plot of standardized residuals for the linear mixed-effects model of depression (DASS-21) in the low-intensity intervention group (Group 2).


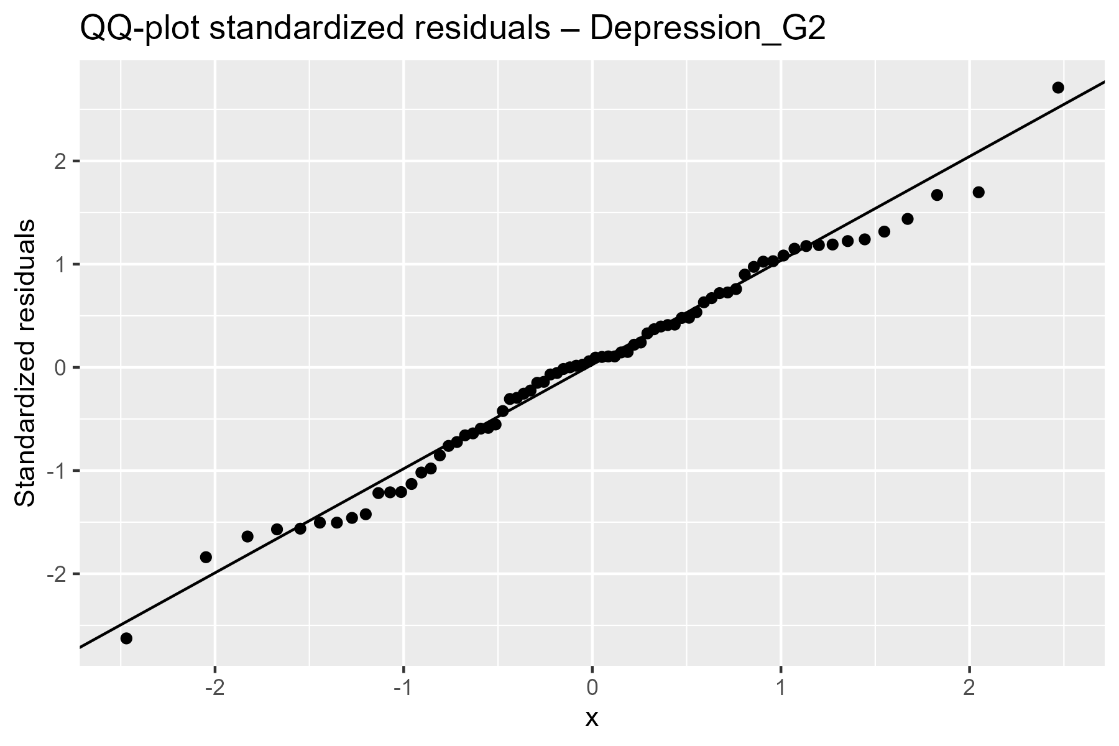


*Residuals follow the theoretical normal distribution with no evidence of extreme outliers.*

**Figure S4.** Residuals versus fitted values for the linear mixed-effects model of depression (DASS-21) in the low-intensity intervention group (Group 2).


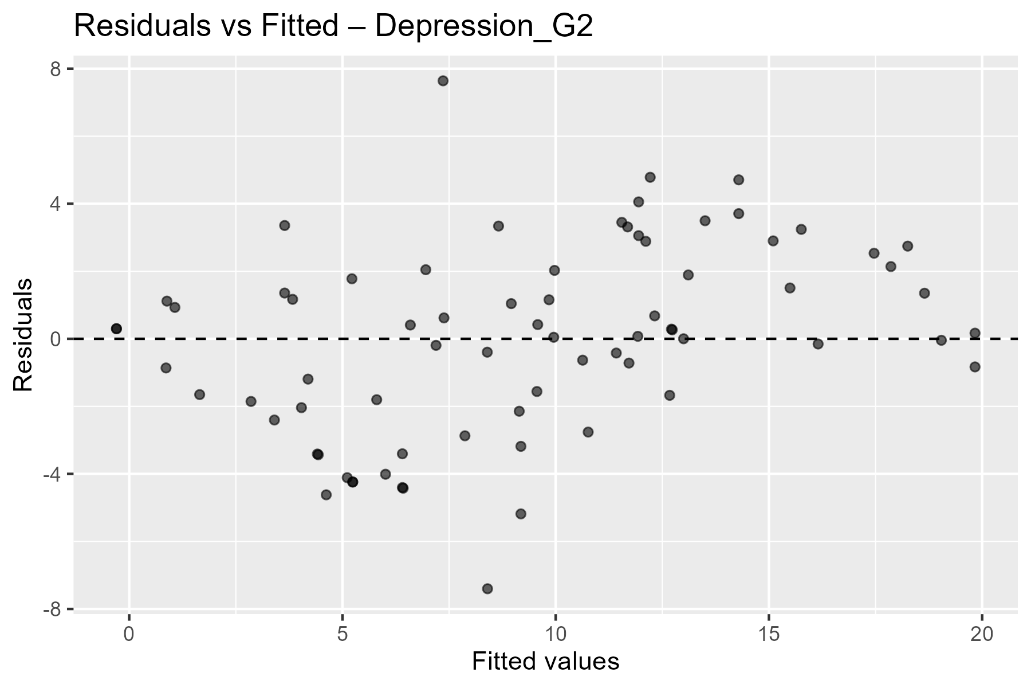


*The dispersion of residuals remains approximately constant across fitted values.*

**Figure S5.** QQ-plot of standardized residuals for the linear mixed-effects model of anxiety (DASS-21) in the high-intensity intervention group (Group 1).


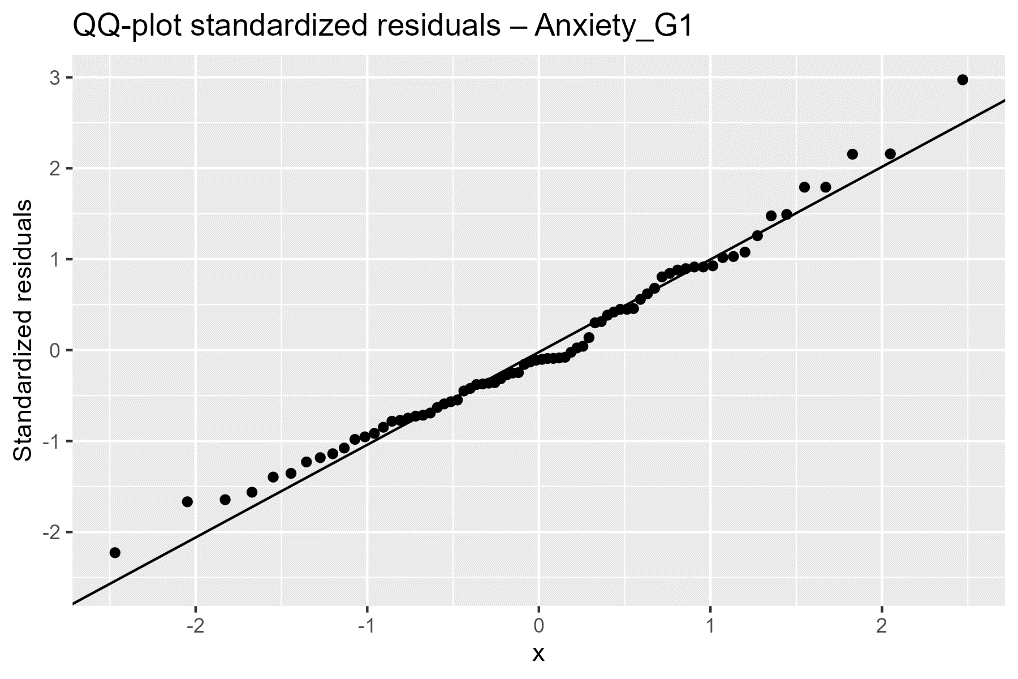


*The distribution of standardized residuals closely follows the normal reference line.*

**Figure S6.** Residuals versus fitted values for the linear mixed-effects model of anxiety (DASS-21) in the high-intensity intervention group (Group 1).


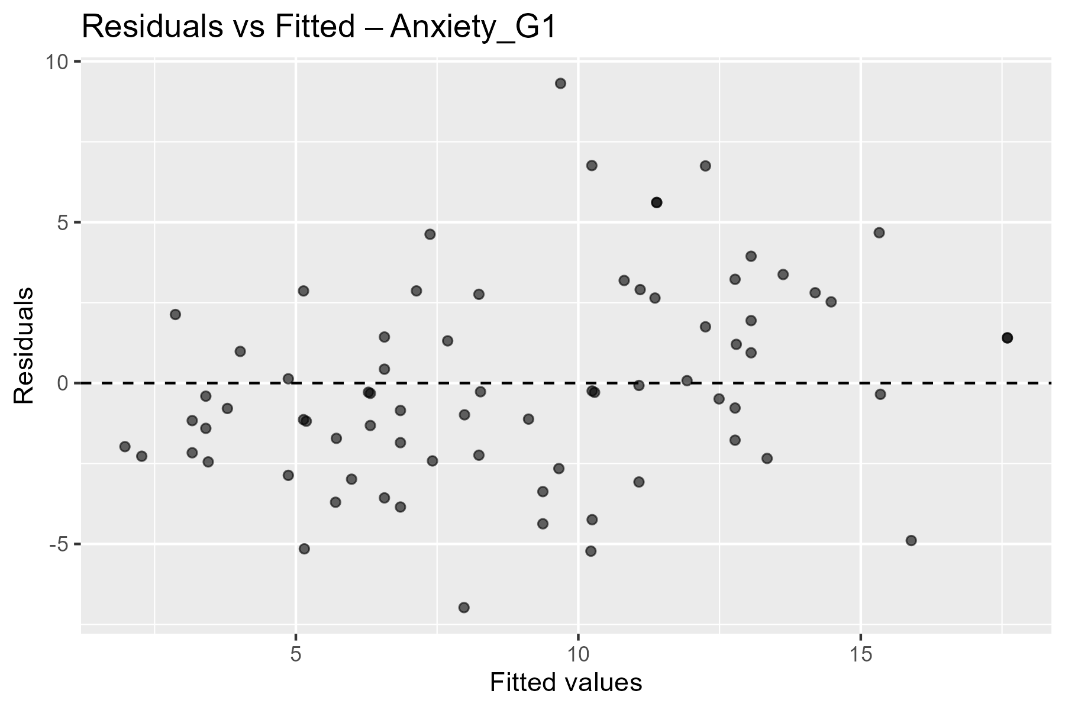


*No systematic patterns or heteroscedasticity are observed.*

**Figure S7.** QQ-plot of standardized residuals for the linear mixed-effects model of anxiety (DASS-21) in the low-intensity intervention group (Group 2).


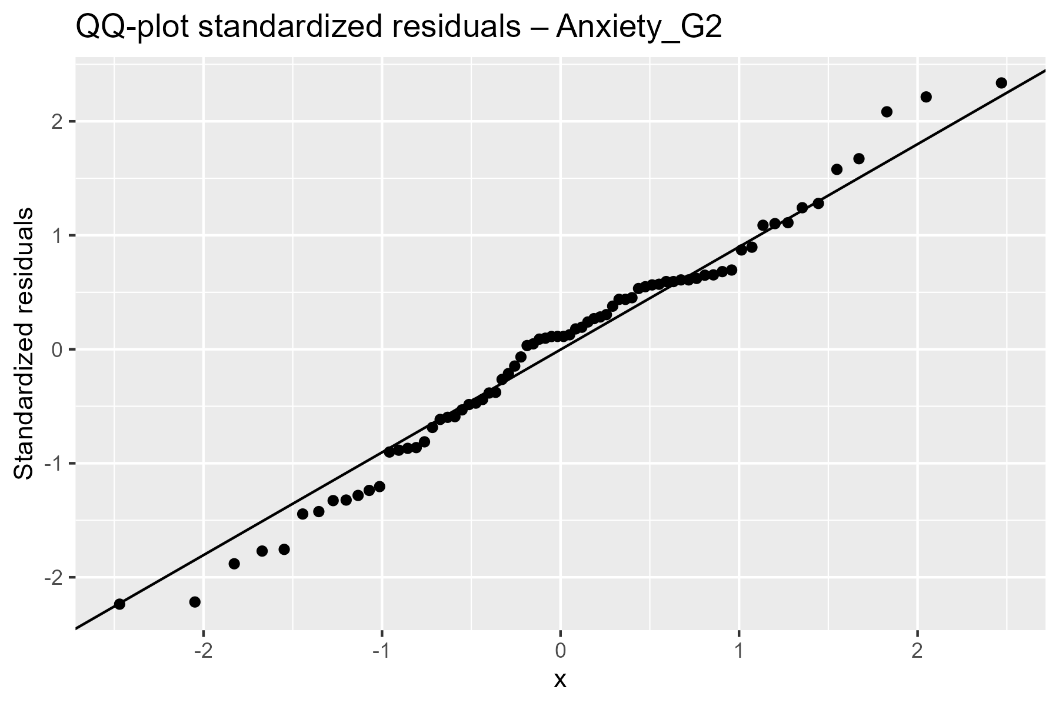


*Residuals show approximate normality, with minor deviations at the distribution tails.*

**Figure S8.** Residuals versus fitted values for the linear mixed-effects model of anxiety (DASS-21) in the low-intensity intervention group (Group 2).


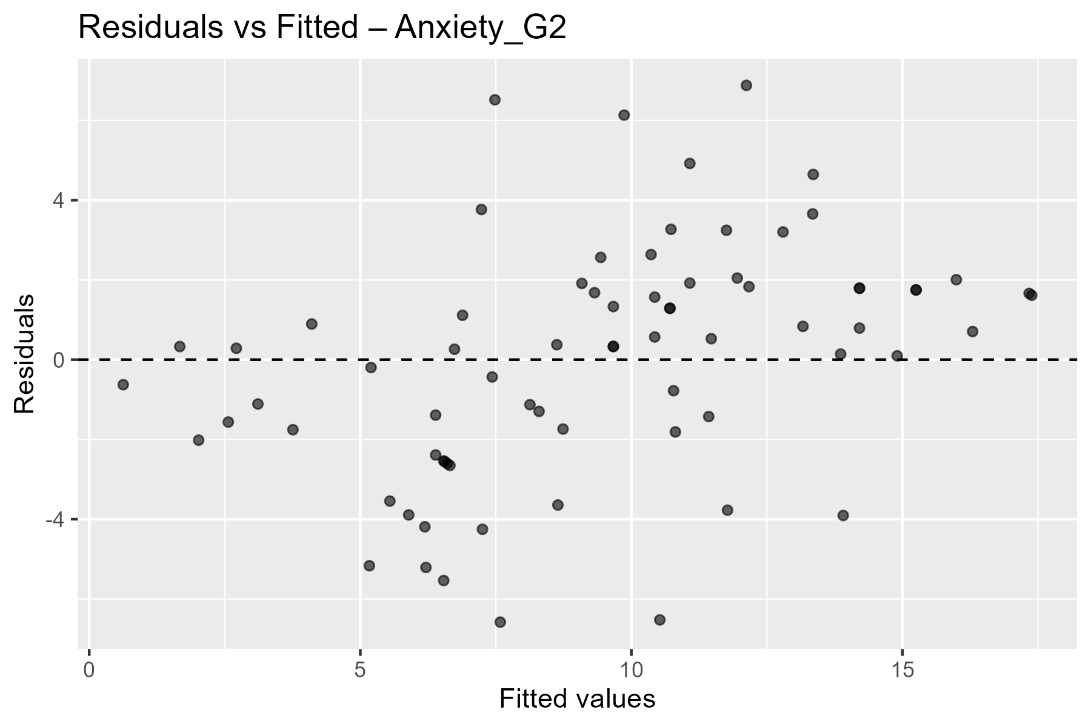


*Residual variance appears stable across the range of fitted values.*

**Figure S9.** QQ-plot of standardized residuals for the linear mixed-effects model of stress (DASS-21) in the high-intensity intervention group (Group 1).


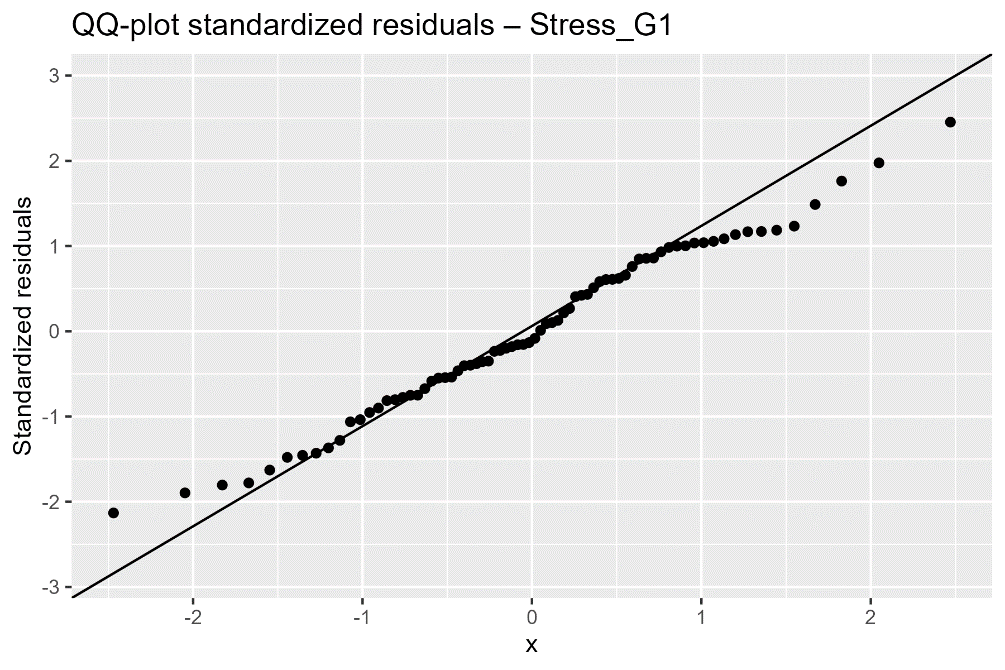


*The residual distribution is consistent with the assumption of normality.*

**Figure S10.** Residuals versus fitted values for the linear mixed-effects model of stress (DASS-21) in the high-intensity intervention group (Group 1).


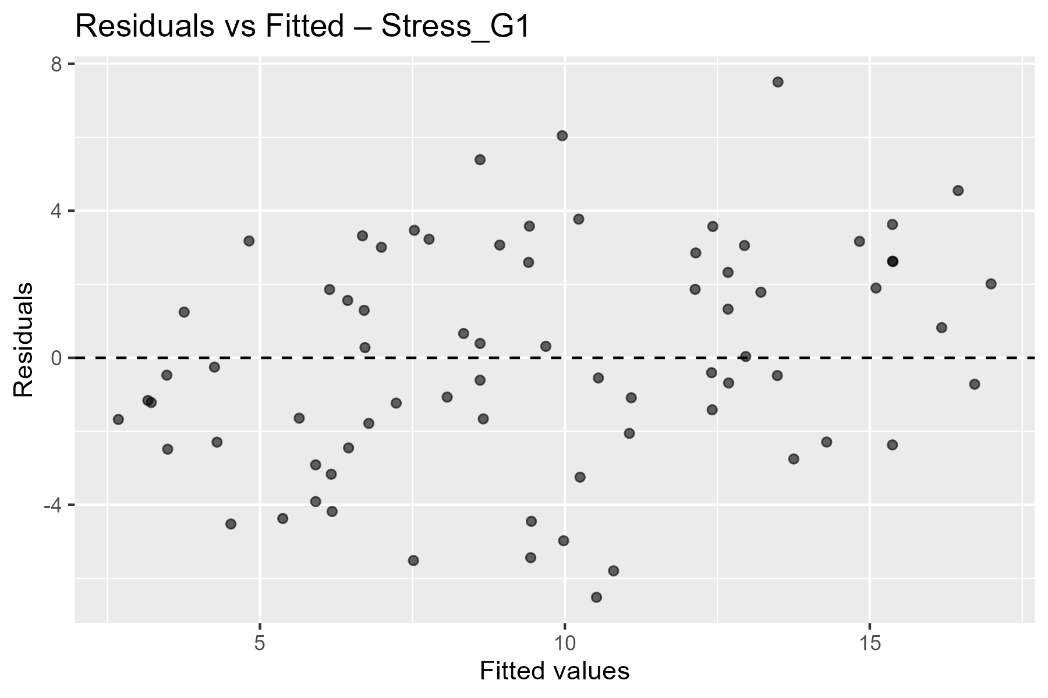


*Residuals are symmetrically distributed around zero with no evident heteroscedasticity.*

**Figure S11.** QQ-plot of standardized residuals for the linear mixed-effects model of stress (DASS-21) in the low-intensity intervention group (Group 2).


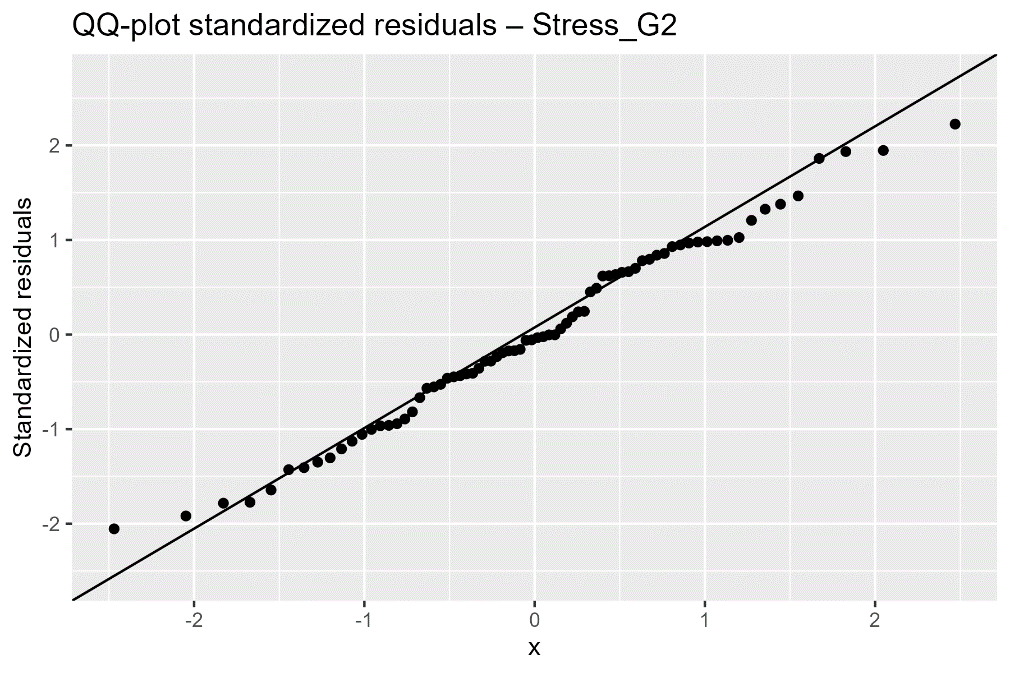


*Standardized residuals closely align with the theoretical normal distribution.*

**Figure S12.** Residuals versus fitted values for the linear mixed-effects model of stress (DASS-21) in the low-intensity intervention group (Group 2).


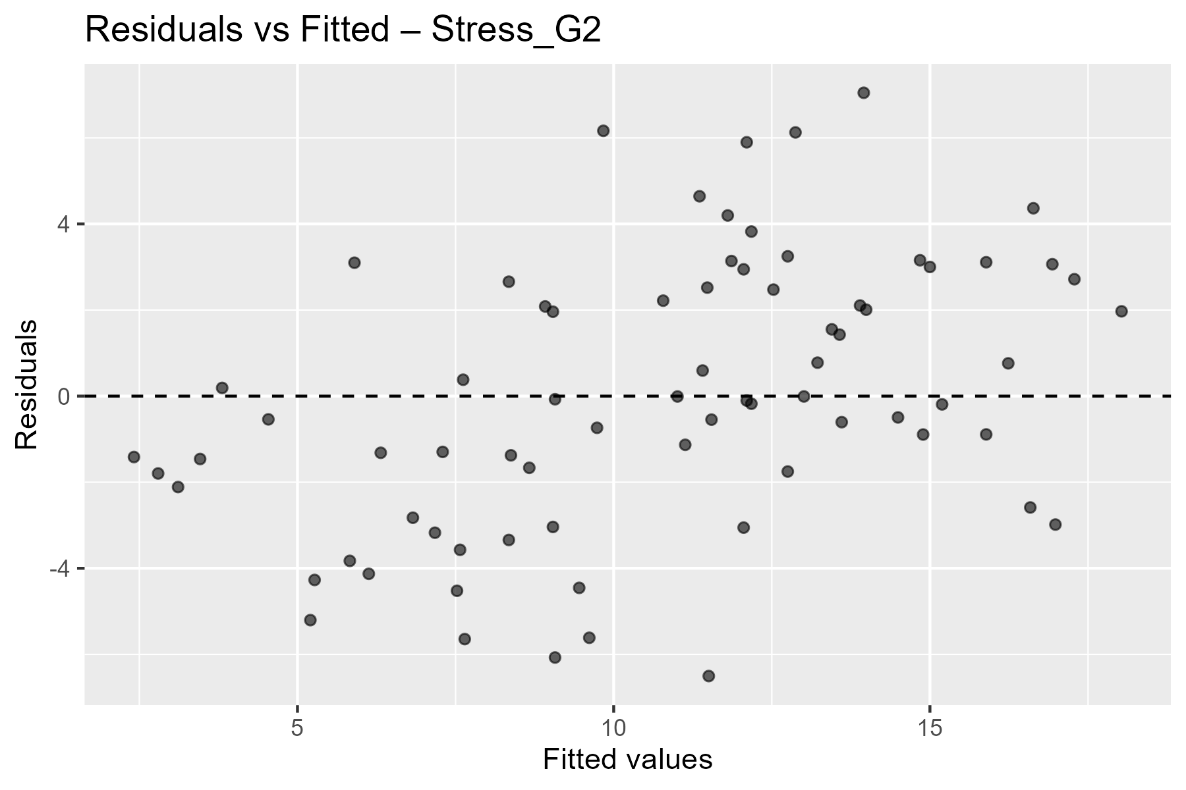


*The absence of structured patterns supports the adequacy of the linear mixed-effects model.*
