# Supplementary material for: High-frequency vs. low-frequency MIDI-assisted group music therapy in psychiatric inpatients: A randomized controlled trial
Source: PLoS One. 2026 Apr 17;21(4):e0317950. doi: 10.1371/journal.pone.0317950 (PMC13089878; doi:10.1371/journal.pone.0317950)
Supplement: S3 Appendix — (PDF) [file pone.0317950.s003.pdf]

Research protocol: part 1

Project summary

Introduction

Music therapy is an evidence-based clinical intervention that has demonstrated efficacy in reducing symptoms like anxiety and depression and in providing neuromodulation that promotes well-being. Few studies evaluate its effectiveness in hospital settings, and the frequency of interventions varies widely, with most interventions being scheduled once per week. Since hospital stays for patients with mental disorders are progressively shorter, it is essential to adapt interventions accordingly and test the effectiveness of music therapy in various frequency schedules.

Materials and Methods

This project will be a randomized clinical trial with two groups: a high-frequency music therapy intervention group (5 sessions/week) and a low-frequency control group (1 session/week). Patients with mental disorders under in-hospital management will be included in the study. The primary outcomes to be measured will be symptoms on the Depression/Anxiety/Stress Scale (DASS-21), while secondary outcomes will be assessed using a questionnaire for the impact of music therapy sessions on adult patients (CISMA, by its Spanish acronym) and a single question on global life satisfaction.

Key Words:

Sensory art therapies, Music therapy, Music, Colombia, Depression, Anxiety, Psychological stress, Psychological well-being.

General information

- **Protocol title:** High-Frequency vs. Low-Frequency Music Therapy in Hospitalized Patients with Mental Disorders: A Randomized Controlled Trial

- **Name and address of the funder:** Dr Manuel Esteban Cardena - Carrera 53 #134 Bogotá. Colombia, City: Bogota D.C, Country: Colombia.
- **Name and title of the investigator(s):** Manuel-F Esteban-Cárdenas1, Eugenio Ferro2, Ana-María Gómez4, Carlos Torres3, Adrián-N Hidalgo-Valbuena5.
    1. Physician and Psychiatry Resident ICSN - Clínica Montserrat - Hospital Universitario, Universidad El Bosque, Bogotá, Colombia.
    2. Psychiatrist and Research Director of ICSN-Clínica Montserrat - Hospital Universitario, Universidad El Bosque, Bogotá, Colombia.
    3. Physician and Researcher at ICSN - Clínica Montserrat - Hospital Universitario, Bogotá, Colombia.
    4. Psychologist and Researcher of the ICSN - Clínica Montserrat - Hospital Universitario, Bogotá, Colombia.
    5. Musician and Music Therapist of the University of La Rioja.
- **Organisation:** Instituto Colombiano del Sistema Nervioso-Clinica Montserrat, Address: Calle 134 #17-71, Bogotá D.C., Colombia, Tel: +57-6012596000

Rationale & background information

The relationship between music and health has been widely studied (1). It has evolved as a complementary intervention for the treatment of mental illnesses and disorders. The American Music Therapy Association (AMTA) defines music therapy as the clinical and evidence-based use of musical interventions to achieve specific therapeutic goals in the treatment of various physical and psychological disorders (2). That are based on the premise that emotional music influence, cognitive and physical aspects of human well-being, stimulating areas of the brain linked to emotions, memory and behavior (2).

Music therapy, in addition to demonstrating benefits in the general wellbeing of people, has been shown to be used as a complementary therapeutic intervention in the treatment of various physical and mental health conditions (3,4). Its versatility allows it to be applied in different settings, populations and pathologies (5-7).

It has been demonstrated that music therapy produces modulation of neurochemical systems, promoting relaxation and well-being (6). In addition, it promotes neuroplasticity and release of neurotransmitters such as dopamine and serotonin, related to pleasure, motivation and well-being (8). Both passive, and active participation in its creation have shown positive effects on cognitive and psychosocial functioning (5) as well as reducing pain and anxiety (9).

Previous studies support music therapy as an effective non-pharmacological intervention for emotional regulation, significantly reducing symptoms of anxiety and depression in different populations, including patients with chronic diseases such as those on hemodialysis (10), people with dementia (11) and psychiatric patients, highlighting its impact on improving mood and self-expression (12). It has also been reported that the combination with Progressive Muscle Relaxation (PMR) is effective in reducing anxiety, depression and stress in patients with breast cancer and gynecological cancer during chemotherapy, also improving the life satisfaction of these patients (13).

However, although music therapy has demonstrated its effectiveness, important questions remain regarding the optimal frequency and duration of the intervention, especially in patients with mental disorders in hospital treatment.

Most music therapy intervention studies with clinical populations contemplate sessions with a weekly frequency and over a relatively long period of several weeks to months. However, psychiatric hospitalizations are currently much shorter, lasting about 10 to 12 days. For this reason, it is necessary to test music therapy interventions that can be administered for short periods, to be adapted as a complementary treatment to psychiatric hospitalization. To the best of our knowledge there is no evidence on the benefit of music therapy sessions more frequently than once a week in a hospital setting in mental patients.

The aim of the present study was to evaluate the effects of high frequency music therapy (five sessions per week) compared to low frequency (one session per week) in patients with mental disorders. Our hypothesis is that patients receiving higher frequency of sessions might have better effects on symptoms of anxiety, depression, stress, and life satisfaction.

References (of literature cited in preceding sections)

1. De Sousa A. The role of music therapy in psychiatry. Altern Ther Health Med. 2005;11(6).

2. American Music Therapy Association®. About Music Therapy and AMTA _ American Music Therapy Association (AMTA) [Internet]. [cited 2024 Oct 8]. Available from: https://www.musictherapy.org/about/

3. Tao A, Ho KHM, Yang C, Chan HYL. Effects of non-pharmacological interventions on psychological outcomes among older people with frailty: A systematic review and meta-analysis. Vol. 140, International Journal of Nursing Studies. 2023.

4. Huang J, Yuan X, Zhang N, Qiu H, Chen X. Music therapy in adults with copd. Vol. 66, Respiratory Care. 2021.

5. Viola E, Martorana M, Airoldi C, Meini C, Ceriotti D, De Vito M, et al. The role of music in promoting health and wellbeing: a systematic review and meta-analysis. Eur J Public Health [Internet]. 2023 Aug 1 [cited 2024 Oct 2];33(4):738–45. Available from: https://pubmed.ncbi.nlm.nih.gov/37322515/

6. Lee H, Ko B. Effects of Music-Based Interventions on Motor and Non-Motor Symptoms in Patients with Parkinson's Disease: A Systematic Review and Meta-Analysis. Vol. 20, International Journal of Environmental Research and Public Health. 2023.

7. Yue W, Han X, Luo J, Zeng Z, Yang M. Effect of music therapy on preterm infants in neonatal intensive care unit: Systematic review and meta-analysis of randomized controlled trials. Vol. 77, Journal of Advanced Nursing. 2021.

8. Sutton J, Backer jos. The music in music therapy psychodynamic music therapy in Europe. British Journal of Music Therapy. 2015;29(2):39–42.

9. Santiváñez-Acosta R, Tapia-López E de las N, Santero M. Music Therapy in Pain and Anxiety Management during Labor: A Systematic Review and Meta-Analysis. Medicina (Kaunas) [Internet]. 2020 Oct 1 [cited 2024 Oct 2];56(10):1–11. Available from: https://pubmed.ncbi.nlm.nih.gov/33050409/

10. Soliva MS, López CC, Salvador IR, Ramón RO, Coca JV, Maset RG, et al. The effectiveness of live music in reducing anxiety and depression among patients undergoing haemodialysis. A randomised controlled pilot study. PLoS One [Internet].

2024 Aug 1 [cited 2024 Oct 2];19(8). Available from: https://pubmed.ncbi.nlm.nih.gov/39186740/

11. Zhang J, Yu Z, Zhang N, Zhao W, Wei B, He R, et al. Does music intervention relieve depression or anxiety in people living with dementia? A systematic review and meta-analysis. Vol. 27, Aging and Mental Health. Routledge; 2023. p. 1864–75.

12. Choi AN, Lee MS, Lim HJ. Effects of group music intervention on depression, anxiety, and relationships in psychiatric patients: A pilot study. Journal of Alternative and Complementary Medicine. 2008 Jun 1;14(5):567–70.

13. Liao J, Wu Y, Zhao Y, Zhao Y chen, Zhang X, Zhao N, et al. Progressive Muscle Relaxation Combined with Chinese Medicine Five-Element Music on Depression for Cancer Patients: A Randomized Controlled Trial. Chin J Integr Med. 2018;24(5).

14. Antony MM, Cox BJ, Enns MW, Bieling PJ, Swinson RP. Psychometric properties of the 42-item and 21-item versions of the Depression Anxiety Stress Scales in clinical groups and a community sample. Psychol Assess. 1998;10(2).

15. Valverde MR, Hernández López M, Ruiz Jiménez F, Barnes-Holmes D, Morales JF, Papini M, et al. InternatIonal Journal of Psychology & PsychologIcal theraPy edItor revIewIng edItors assocIate edItors assIstant edItors ManagIng edItor edItorIal offIce/secretaría de edIcIón. InternatIonal Journal of Psychology & PsychologIcal theraPy [Internet]. 2017;17(1). Available from: http://www.ijpsy.comhttp://www.ijpsy.com

16. Vargas-Olano MO, Cárdenas-Ojeda SP, Herrera -Delgado C. Un recurso para atención primaria de la salud mental. DASS-21, propiedades psicométricas. Revista Peruana de Investigación en Salud. 2022 Jul 30;6(3):141–8.

17. Vercher IB, Soler AA, Ferrari KD. CUESTIONARIO CISMA - CUESTIONARIO DEL IMPACTO DE LAS SESIONES DE MUSICOTERAPIA EN PACIENTES ADULTOS. Brazilian Journal of Music Therapy. 2023;

18. Lauri Korajlija A, Mihaljevic I, Jokic-Begic N. Single-Item Life Satisfaction Measurement. Socijalna psihijatrija [Internet]. 2019 Dec 30;47(4):449–69. Available

from:
https://www.socijalnapsihijatrija.com/en/social-psychiatry/47/47-4/single-item-life-satisfaction-measurement/

19. Asamblea Médica Mundial. Declaración de Helsinki de la Asociación Médica Mundial Recomendaciones para guiar a los médicos en la investigación biomédica en personas. Vols. 1–4, Conamed. 2013.

20. MINISTERIO DE SALUD. RESOLUCION No 008430 DE 1993 (4 DE OCTUBRE DE 1993). REPUBLICA DE COLOMBIA 1993.

21. Aalbers S, Fusar-Poli L, Freeman RE, Spreen M, Ket JCF, Vink AC, et al. Music therapy for depression. Vol. 2017, Cochrane Database of Systematic Reviews. John Wiley and Sons Ltd; 2017.

22. Lee H, Ko B. Effects of Music-Based Interventions on Motor and Non-Motor Symptoms in Patients with Parkinson's Disease: A Systematic Review and Meta-Analysis. Vol. 20, International Journal of Environmental Research and Public Health. MDPI; 2023.

23. Colin C, Prince V, Bensoussan JL, Picot MC. Music therapy for health workers to reduce stress, mental workload and anxiety: a systematic review. J Public Health (Oxf). 2023 Sep 1;45(3):e532–41.

24. Zhong D, Cheng H, Pan Z, Liu Y, Liu P, Li J, et al. The safety and effectiveness of music medicine as an intervention for depression: A systematic evaluation and re-evaluation. Brain Behav [Internet]. 2024 Sep 1 [cited 2024 Oct 2];14(9). Available from: https://pubmed.ncbi.nlm.nih.gov/39262200/

25. Zhou K, Li X, Li J, Liu M, Dang S, Wang D, et al. A clinical randomized controlled trial of music therapy and progressive muscle relaxation training in female breast cancer patients after radical mastectomy: Results on depression, anxiety and length of hospital stay. European Journal of Oncology Nursing. 2015;19(1).

26. Nguyen KT, Hoang HTX, Bui Q V., Chan DNS, Choi KC, Chan CWH. Effects of music intervention combined with progressive muscle relaxation on anxiety, depression, stress and quality of life among women with cancer receiving

chemotherapy: A pilot randomized controlled trial. PLoS One [Internet]. 2023 Nov 1 [cited 2024 Oct 2];18(11). Available from: https://pubmed.ncbi.nlm.nih.gov/37922279/

27. Kacem I, Kahloul M, El Arem S, Ayachi S, Hafsia M, Maoua M, et al. Effects of music therapy on occupational stress and burn-out risk of operating room staff. Libyan Journal of Medicine. 2020 Jan 1;15(1).

28. Gallego-Gómez JI, Balanza S, Leal-Llopis J, García-Méndez JA, Oliva-Pérez J, Doménech-Tortosa J, et al. Effectiveness of music therapy and progressive muscle relaxation in reducing stress before exams and improving academic performance in Nursing students: A randomized trial. Nurse Educ Today. 2020 Jan 1;84.

29. Liu YH, Lee CCS, Yu CH, Chen CH. Effects of music listening on stress, anxiety, and sleep quality for sleep-disturbed pregnant women. Women Health. 2016 Apr 2;56(3):296–311.

30. Bradt J, Dileo C, Myers-Coffman K, Biondo J. Music interventions for improving psychological and physical outcomes in people with cancer. Cochrane Database Syst Rev [Internet]. 2021 Oct 12 [cited 2024 Oct 2];10(10). Available from: https://pubmed.ncbi.nlm.nih.gov/34637527/

31. Umbrello M, Sorrenti T, Mistraletti G, Formenti P, Chiumello D, Terzoni S. Music therapy reduces stress and anxiety in critically ill patients: A systematic review of randomized clinical trials. Vol. 85, Minerva Anestesiologica. Edizioni Minerva Medica; 2019. p. 886–98.

32. Han L, Li JP, Sit JWH, Chung L, Jiao ZY, Ma WG. Effects of music intervention on physiological stress response and anxiety level of mechanically ventilated patients in China: A randomised controlled trial. J Clin Nurs. 2010;19(7–8):978–87.

Objectives and goals of the study

General Objective:

- To describe the response to high-intensity music therapy in hospitalized patients at the Montserrat Clinic.

Specific Objectives:

- To describe the sociodemographic and clinical characteristics of patients hospitalized with mental illness at Clínica Montserrat.
- To compare the levels of anxiety, depression, stress, levels of general life satisfaction and perception of the impact of music therapy. Before and after low and high intensity music therapy in patients hospitalized at Clínica Montserrat.
- To explore possible associations between sociodemographic and clinical characteristics of patients and their response to low-intensity versus high-intensity music therapy.

Study design

The study will be a randomized controlled clinical trial. Participants will be randomly assigned in a 1:1 ratio to either the high-frequency music therapy intervention group or the low-frequency control group. Randomization will be performed using random number generation. The high-frequency intervention group will receive five music therapy sessions over one week of inpatient treatment, at a rate of one session per day for five consecutive days. The low-frequency control group will receive one music therapy session in one week of inpatient treatment.

The primary outcome will assess symptoms of depression, anxiety, and stress during inpatient treatment. Secondary outcomes will include the perceived impact of the music therapy session on well-being and self-reported life satisfaction.

Participants

Inclusion criteria:

- Individuals over 18 years of age.

- Psychiatric hospitalization of at least 48 hours at recruitment.
- Pharmacological treatment with medication adjustments in the last two weeks.

Exclusion criteria:

- Prior participation in music therapy programs.
- Primary hospitalization diagnosis of abstinence syndrome or substance dependence.
- Hospitalization duration exceeding six days at the time of selection.

A total of 106 patients undergoing inpatient treatment are expected to be identified over a 32-day period between August and September 2024. A sample of 91 participants is projected to accept participation and will be randomized to either the high-frequency intervention group or the low-frequency control group.

Methodology

Study Type: Randomized clinical trial with a concurrent comparative group.

Population and Sample

Population: Patients hospitalized in intermediate or general psychiatry units at Clínica Montserrat.

Sample Size: Based on relevant studies, especially that of Cantekin & Tan (2013) on music therapy's impact on stress reduction in hemodialysis patients, the response rate to music therapy is estimated to be significant in stress reduction. Although exact percentages are not provided, a statistically significant reduction in stress scores ($p < 0.01$) suggests considerable patient response. The sample size will be calculated to ensure sufficient power for detecting similar effects.

Inclusion Criteria:

- Adults 18 years or older at recruitment.
- Patients undergoing pharmacological adjustments within the last two weeks.
- Patients hospitalized for less than 48 hours.

Exclusion Criteria:

- Previous participation in music therapy.
- Primary diagnosis of substance withdrawal or dependence.
- Hospitalization duration exceeding six days before the start of the first music therapy session.

Variables

Sociodemographic data will be collected, including age, gender, educational level, and clinical information such as hospitalization diagnosis, levels of anxiety, depression, stress, general discomfort, life satisfaction perception, intervention-related data like expectations regarding music therapy, and the weekly intensity of music therapy received.

Dependent Variables:

- Intensity of Music Therapy:
    - Low intensity
    - High intensity

Independent Variables:

- Distress levels for anxiety symptoms.
- Discomfort levels for depressive symptoms.
- Discomfort levels for stress.
- General discomfort levels.
- Life satisfaction levels.

Sociodemographic Variables:

- Age
- Gender
- Educational level

Clinical Variables:

- Mental illness diagnosis

- Symptom severity

Variables Related to Music Therapy:

- Expectations of music therapy intervention
- Impact of music therapy
- Weekly intensity of music therapy

Randomization

Prior randomization of 100 subject records will be conducted using Excel, generating random numbers from zero to one with the “=RAND()” function and using the formula “=IF(Cell<0.5, 'A', 'B')” to assign patients to either Group A or Group B based on values below or above 0.5.

Selection of participants for recruitment will involve daily randomized draws through Google Sheets to meet the daily recruitment quota. Recruiters will proceed to explain the study design and obtain informed consent without prior knowledge of the assigned group for each participant.

Each participant will be informed of the measurement methods, including pre- and post-intervention evaluations using scales and tests for different variables. All subjects will participate in both high- and low-intensity intervention modes, ensuring no potential placebo bias given the non-concealable intervention intensity.

After daily recruitment, lists for the next session will be prepared, revealing the group of each participant by registration number.

Safety Considerations

To mitigate risks associated with scale administration and non-participation in music therapy, at least one healthcare professional will attend each session, alongside a certified music therapist.

Monitoring: Clinical and form-based assessments will be conducted throughout the intervention week, with follow-up the following week by health personnel to monitor for any adverse effects.

Data Management and Statistical Analysis

The sample size will be calculated with a power of 85% and a bilateral significance level of 0.05, aiming to detect a mean difference of 4 between experimental and control groups, with an estimated standard deviation of 5. A minimum sample size of 60 subjects (30 per group) will be targeted, with 35 per group to account for possible dropouts.

Data analysis will use Jamovi software, version 2.6 (2024). Descriptive analysis will characterize the sample, including central tendency and dispersion measures, and the normality of continuous variables will be assessed using the Shapiro-Wilk test. Differences between intervention and control groups will be determined using the Mann-Whitney U test for non-normally distributed data, and correlation analyses will use Spearman's nonparametric test where applicable. The analysis will include evaluations of sociodemographic and initial characterization variables, complemented by a correlation matrix for primary variables and outcomes.

Quality assurance

Both the research protocol and the informed consent were approved by an independent research ethics committee (CEI Campo Abierto Ltda, approval certificate No. 209, July 2024). All study participants signed the informed consent document before performing any research procedures.

Expected results of the study

1. **Contribution to the Advancement of Knowledge**

This study will contribute significantly to the field of music therapy in psychiatric settings, particularly in hospitalized patients with mental health disorders. The observed difference in stress reduction between the high and low frequency groups indicates that the intensity of music therapy may be a key factor in treatment efficacy. The association between positive expectations towards music therapy and greater symptom reduction offers a novel perspective on the importance of the psychological component in non-pharmacological treatments, suggesting that patient predisposition may influence therapeutic outcomes.

2. **Application of the Results in Health Care**

The results of the study could encourage the integration of high-frequency music therapy sessions as a regular intervention in the treatment of hospitalized psychiatric patients, especially for those experiencing elevated levels of stress. This could improve current approaches to stress and anxiety management in this population group, helping to reduce reliance on anxiolytic or antidepressant medications in certain cases.

The observation that both groups experienced improvements in anxiety, depression, and life satisfaction supports the inclusion of music therapy, regardless of frequency, as a valuable intervention in inpatient treatment programs.

3. **Impact on Health Systems**

Incorporating music therapy into inpatient programs for patients with mental disorders may offer a cost-effective therapeutic alternative and potentially reduce hospital stay or the need for additional interventions, which could optimize the use of resources in the health care system.

The use of an intensity-based approach to music therapy provides a basis for health systems to implement programs tailored to the needs and responsiveness of patients, thus maximizing the effectiveness of mental health interventions.

4. **Potential Health Policy Implications**

This study could serve as a reference to guide mental health policies that include complementary therapies such as music therapy in psychiatric care protocols, recognizing the importance of non-invasive therapies in improving patients' well-being.

Evidence of the efficacy of music therapy in reducing stress and improving well-being could motivate policy makers to include and fund such interventions in hospitals and psychiatric facilities, promoting a holistic approach to mental health treatment.

5. **Publications and Knowledge Dissemination**

The findings will be published in academic journals to inform the scientific community about the efficacy of different intensities of music therapy in psychiatric patients. In addition, it is expected that these results will be shared at mental health conferences and workshops so that professionals in the field can implement these approaches in their clinical practices.

Data on the relationship between expectations and intervention outcomes may provide a basis for future research on how psychological factors influence the effectiveness of nonpharmacological therapies, promoting a biopsychosocial approach to psychiatric care.

Dissemination of results and publication policy

Dissemination of the results will be carried out at different levels in order to maximize the impact of the study and benefit the community, participants and public policy makers.

- Scientific dissemination: The results of the study will be published in high impact scientific journals in the field of music therapy and psychiatry, such as PLOS ONE or similar open access journals, to ensure accessibility of the findings to researchers and clinicians.
- Dissemination to the community: These will be planned with the clinical staff of the institution. These meetings will allow socializing the findings and answering questions, promoting a better understanding of the potential benefits of music therapy.
- Dissemination to policy makers: If the findings suggest significant benefits, executive summaries will be generated and presentations will be made to local and national agencies responsible for mental health policy. The intention is to provide evidence to support the inclusion of music therapy in mental health programs.

Publication Policy

To ensure clarity and transparency in the publication of results, the following guidelines will be established:

- Responsibility for publication: The research team will take the lead in the publication of findings. Decisions regarding the choice of journals and content of manuscripts will be made in consensus by the research team.
- Acknowledgement of contributions: All investigators and collaborators who have contributed substantially to the design, development, analysis, or interpretation of data will be acknowledged in publications, following international standards for authorship attribution and acknowledgement of contributions. In addition, the institutions and individuals who provided resources or supported the study will be mentioned and thanked.
- Access to the results: It will be guaranteed that the publications resulting from the study will be open access, in order to promote the wide availability of the knowledge generated, in line with the commitment to transparency and community benefit.

The recruitment process will involve a convenience sample of patients hospitalized at Clínica Montserrat who meet the inclusion and exclusion criteria over a projected period of forty-two days. Given that the average hospitalization duration is approximately ten days, the recruitment schedule is organized to allow each participant to access the intervention prior to their anticipated discharge.

Daily recruitment will begin with a review of the patient census, listing each patient's hospitalization days. This review will identify eligible patients for recruitment each day based on their hospitalization timeline. Selected patients will be prioritized, starting with those furthest along in their hospitalization (ideally those on their ninth day) and continuing with those who have fewer days, ensuring they meet the criteria to receive the intervention in a timely manner.

On each recruitment day, eligible patients will be actively sought within the clinic to provide them with information about the study and obtain informed consent from those willing to participate. Daily recruitment will yield a count of recruited patients and a record of those who decline, to adjust the next day’s recruitment targets accordingly.

Priority will initially be given to patients with longer hospitalization durations to maximize intervention exposure. As recruitment progresses and patients with extended stays are enrolled, focus will gradually shift toward those with shorter hospitalizations. This approach is designed to maintain consistent group sizes for the intervention sessions, facilitating robust and enriched group therapy sessions throughout the recruitment period.

This systematic and phased recruitment strategy is intended to ensure timely enrollment and intervention exposure for each participant while adhering to the study’s target sample size and optimizing group therapy dynamics.

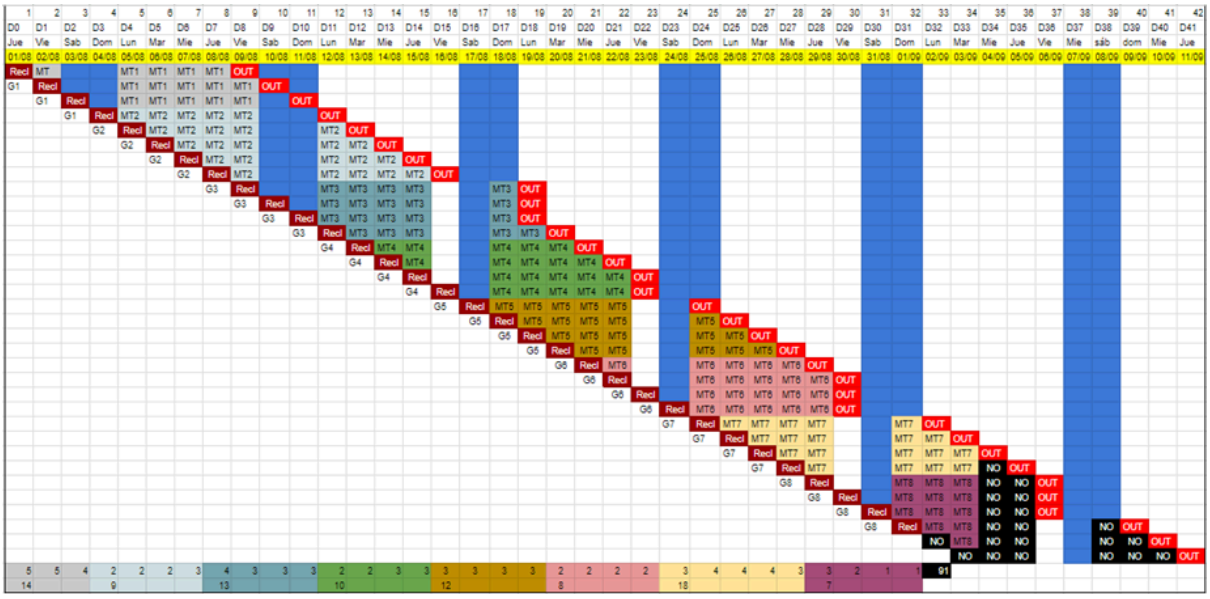

Problems anticipated

The use of self-reports and observation by the music therapist may generate biases of social desirability and subjectivity, affecting the validity of the results. Although validated scales such as the DASS-21 and the global life satisfaction scale are used, the CISMA scale is still under development, limiting the robustness of the measurements. The absence of a blinded control group could also introduce expectation bias, especially in those receiving a less intensive intervention. Additionally, the low diversity of psychiatric diagnoses limits the generalizability of the results. It is suggested that future research include additional objective measures and a more diverse sample to strengthen the validity and applicability of these findings.

Project management

- Manuel F Esteban Cárdenas: Conceptualization, Data Curation, Funding Acquisition, Investigation, Methodology, Project Administration, Writing – Review & Editing, Resources
- Eugenio Ferro: Formal Analysis, Methodology, Software, Supervision, Writing – Review & Editing
- Ana María Gómez: Investigation, Visualization, Writing – Original Draft Preparation
- Carlos Torres: Formal Analysis, Software, Validation, Writing – Original Draft Preparation
- Adrián N Hidalgo Valbuena: Investigation, Resources

Ethics

The present work was governed by the biomedical research recommendations of the Declaration of Helsinki of the World Medical Association, as well as the related regulations in the Colombian legislation in force according to Resolution 8430 of 1993 (Technical and administrative scientific norms for health research) (Ministry of Health, 1 1993). On the other hand, according to Resolution No. 008430 OF 1993 (OCTOBER 4, 1993) of the Ministry of Health, this research was considered to be of minimal risk due to the application of common procedures (psychological examinations) to the selected sample. The procedures in the research were carried out by qualified and competent professionals in this type of intervention from a clinical perspective, the researchers did not face conflicts of interest.

The principle of autonomy of the individual to whom participation in the study was proposed was respected, because the decision to participate was free and voluntary and there was no involvement in the treatment by the decision not to participate. For the inclusion of a patient in the study, it was mandatory that he/she did so voluntarily and after signing the informed consent, after receiving detailed information about the study and clarification of any doubts by the qualified professional.

The privacy and confidentiality of the data was guaranteed by assigning an identification code in the research records, likewise, the process of anonymization of sensitive data defined in Law 1581 of 2012 was carried out if necessary. Likewise, security measures were established to protect the information collected in the virtual storage platform of google sheets, With limited access to researchers' own emails which allowed limiting access to the database only to the team of researchers and the collection center (Clínica Montserrat). The results were processed with the assigned codes, and at the time of the disclosure of results, it was done in a general way, without identifying any of the participants. The results of the evaluations could be explained to the participants, if they so wished, privately at the end of the study.

To guarantee the principle of beneficence, although no direct individual benefit was given to the participants, in addition to further follow-up on the significant symptoms of their underlying affective pathology, it highlighted the social and scientific value of

the research in terms of contributing to knowledge about characteristics that impacted the patients' response to music therapy.

To ensure nonmaleficence, the investigators who applied the tests took into account the immediate suspension of the study when they noticed any risk or harm to the health of the participating subjects, or discontinued on an individual basis when they noticed a particular risk in a research subject.

Informed consent forms

- Appendix below

Research protocol: part 2

Budget

1. Personnel Costs

This section includes hourly fees for researchers, advisors, and assistants involved in the project, covering the time they dedicate to their specific responsibilities within the study.

Name	Role in the Project	Hours/Week	Cost/Week	Weeks	Total
Manuel F Esteban Cárdenas	Principal Investigator	20	$760,000	15	$11,400,000
Adrián Hidalgo	Music Therapist	5	$700,000	5	$3,500,000
Eugenio Ferro	Thematic Advisor	4	$340,000	15	$5,100,000
Carlos Torres	Methodological Advisor	2	$140,000	10	$1,400,000
Ana María Gómez	Research Assistant	20	$169,560	6	$1,017,360
Total					**$22,417,360**

Justification: Personnel costs cover the work of researchers, advisors, and data collectors, which are essential for the project's execution.

2. Materials and Supplies

These expenses cover the materials necessary to conduct interventions and data collection activities.

Material/Supply	Justification	Unit Cost	Quantity	Subtotal
MIDI Device Rental	Creative MT interventions	$600,000	1	$600,000
Piano and Organet Rental	Conducting directed MT sessions	$1,200,000	1	$1,200,000
Subscription to Bibliographic Databases (UEB)	Access to international sources	$1,500,000	1	$1,500,000
Access to ICSN Bibliographic Material	Key scientific literature access	$1,000,000	1	$1,000,000
Total				**$4,300,000**

Justification: The subscription to bibliographic resources and rental of instruments are essential for carrying out and enhancing music therapy interventions, ensuring a high-quality, evidence-backed approach.

3. Publication Costs

The budget includes costs associated with publishing results in an indexed scientific journal, crucial for the dissemination and validation of the project's findings.

Publication	Justification	Unit Cost (USD)	Exchange Rate	Subtotal
Publication in Indexed Journal	Editorial publication rights	$800	$3,900	$3,120,000
Total				**$3,120,000**

Justification: Publishing in a high-impact scientific journal ensures the dissemination of knowledge generated by the project, contributing to the fields of music therapy and mental health.

Overall Project Budget Summary

Item	Total Cost
Personnel Costs	$22,417,360
Materials and Supplies	$4,300,000
Publication	$3,120,000
**Total Project Cost**	**$29,837,360**

Other support for the project

- Self-funded

Collaboration with other scientists or research institutions

- Instituto Colombiano del Sistema Nervioso-Clínica Montserrat
- Universidad El Bosque

Links to other projects

- Does not Apply.

Curriculum Vitae of investigators

- Appendix below

Other research activities of the investigators

The principal investigator of this study does not participate in other research projects at present, which guarantees that he devotes his time and attention exclusively to the supervision and development of this study. This exclusive dedication allows us to maintain high quality standards and detailed monitoring of compliance with the objectives and procedures established in the protocol.

In addition, this study has been financed entirely self-funded. This ensures that there are no conflicts of interest with external entities and that the decisions in the development of the research are oriented exclusively to the fulfillment of the scientific and ethical objectives of the project, without the influence of sponsors or third party financing.

The absence of other research commitments and the self-funded nature of this study contribute to the independence, impartiality in the execution and analysis of the results, strengthening the validity and reliability of the study.

Financing and insurance

- Does not Apply.
