# Supplementary material for: High-frequency vs. low-frequency MIDI-assisted group music therapy in psychiatric inpatients: A randomized controlled trial
Source: PLoS One. 2026 Apr 17;21(4):e0317950. doi: 10.1371/journal.pone.0317950 (PMC13089878; doi:10.1371/journal.pone.0317950)
Supplement: Attachment — Submitted filename: Rebutal letter_6Jun2025.docx [file pone.0317950.s006.docx]

**Manuscript Title**: "**High-Frequency vs. Low-Frequency Music Therapy in Psychiatric Inpatients: A Randomized Controlled Trial**"

PONE-D-24-55131

Dear Academic Editor and Reviewers,

We sincerely thank you for your thoughtful and constructive comments on our manuscript submitted to *PLOS ONE*. We have carefully reviewed all suggestions and have made the corresponding changes to improve the clarity and scientific rigor of the manuscript.

Below, we provide a detailed point-by-point response to each comment. Reviewer comments are shown in italic, followed by our responses in regular font and a description of changes made to the manuscript. All changes made to the manuscript have been highlighted in the revised version for your convenience.

___________________________________________________________________

**Reviewer 1 – Comment 1:**
*First, I question whether the sample size calculation was conducted correctly. The authors state that they aimed to detect a mean difference of 4 between the experimental and control groups, with an estimated standard deviation of 5. However, it is unclear what this difference of 4 represents. Typically, sample size calculations are based on effect size, which must be explicitly linked to the intervention outcome.*

**Response:**
We appreciate the reviewer’s insightful observation. We have revised the manuscript to clarify the rationale behind our sample size calculation. The originally reported difference of 4 points corresponds to an expected mean difference in outcome scores based on prior literature. However, for clarity and precision, we now express this as an effect size of 0.6, following the findings reported by Zhang et al. (2022), which served as a reference for expected clinical impact in music therapy interventions. Accordingly, the sample size was calculated using a two-tailed test with α = 0.05 and power = 0.80, assuming a 1:1 allocation ratio and a medium effect size (Cohen’s d = 0.6). This yielded a required sample of 30 participants per group. Accounting for an anticipated 20% dropout rate, we aimed to recruit a total of 74 participants (37 per group). The revised text is now included in the Methods section.

**Reviewer 1 – Comment 2:**
*Additionally, the software and statistical model used to determine this number should be specified.*

**Response:**
Thank you for this observation. We have now included the software version used for the sample size calculation in the Methods section. Specifically, the calculation was performed using RStudio (version 2024.09.0+375). The statistical model applied was based on a two-sample t-test assuming equal variances and a two-sided significance level of 0.05.

**Reviewer 1 – Comment 3:**
*One of the inclusion criteria is “pharmacological treatment with medication adjustments.” Given that different medications may be prescribed based on condition severity, they could also influence the effectiveness of the music therapy intervention. Have the authors collected and compared medication data between groups?*

**Response:**
We thank the reviewer for this important observation. We acknowledge that different pharmacological treatments could have influenced participants’ responses to the music therapy intervention. However, medication type and dosage were not systematically collected or compared between groups. This has now been explicitly recognized as a limitation in the revised manuscript. Additionally, we have added a new subsection on study limitations within the Discussion section to address this and other methodological considerations identified during the revision process.

**Reviewer 1 – Comment 4:**

*The authors conducted a preliminary Shapiro-Wilk test for normality, which yielded significant results. Consequently, they opted for non-parametric tests throughout the analysis. However, this choice precludes the use of more robust methods, such as repeated measures analysis. [...] In situations where the deviation from normality is not substantial, a conventional approach can still be used.*

***Reviewer 1 – Comment 5:***

*Additionally, multiple outcomes were analyzed without adjustments for multiple comparison bias. The only significant p-value (0.03) is relatively weak and would likely lose significance with even a mild adjustment. Thus, the evidence supporting the effectiveness of high-frequency music therapy is not strong.*

**Comment 4 & 5 Combined Response:**

Thank you for these important observations. In response, we reanalyzed the data using mixed-effects linear models to account for both fixed effects (time, group, and their interaction) and random effects, while controlling for relevant covariates including patients' diagnostic categories and time. This modeling approach provides greater robustness by accommodating within-subject variability and helps reduce the risk of inflated Type I error due to multiple comparisons.

Although initial Shapiro-Wilk tests indicated non-normality, diagnostic plots showed homoscedastic residuals and no influential outliers, supporting the validity of these models.

Regarding the DASS-21, both groups improved post-intervention. The stress subscale showed a significant group-by-time interaction (p = 0.023), with the five-session group exhibiting a greater reduction (−6.19 points), suggesting a frequency-dependent effect.

In the anxiety subscale, the five-session group had a significant pre-post reduction (−5.41 points, p < 0.001), but the group-by-time interaction was not significant (p = 0.339), indicating similar improvements across groups. Depression scores also improved significantly in both groups, though no significant group-by-time difference was found (p = 0.27). For life satisfaction (SWLS), the five-session group showed a significant post-intervention increase (+11.6 points, p = 0.012), but no significant between-group change was observed.

To assess the subjective perception of change, we used linear mixed models to the selected CISMA items, where we included items 1, 3 and 4 for what we wanted to measure that was emotional changes, where time was incorporated as a covariate. so q Although tests of normality of residuals were significant, the diagnostic plots supported the adequacy of the model. None of the CISMA items showed statistically significant differences between groups after the intervention, and the models explained limited variance, although slight trends in favor of the five-session group were observed.

These results have been incorporated into the revised manuscript. The use of mixed models with covariate adjustment provides a more conservative and informative analysis framework that reduces the likelihood of spurious findings.

**Reviewer 1 – Comment 6:**

*The authors also conducted a series of correlations between expectation levels and various outcomes. However, since expectation levels are not directly related to the intervention itself, this analysis falls outside the study’s scope and does not support the intervention’s effectiveness.*

**Response:**

We appreciate the reviewer’s thoughtful observation regarding the correlation analyses between patients’ expectations and clinical outcomes. We fully agree that expectations represent a distinct construct from the intervention itself, and therefore, any associations identified between expectations and outcomes cannot be interpreted as direct evidence of the intervention’s effectiveness. As noted, significant correlations (e.g., p < 0.05) do not imply causality.

Furthermore, these correlations were not part of the study’s primary experimental design and did not account for key variables such as intervention group or time effects. Thus, they were presented as exploratory findings intended to complement the main results by highlighting possible psychosocial factors associated with changes in clinical scores (e.g., DASS-21), rather than as proof of treatment efficacy. We have clarified this point in the revised manuscript to avoid misinterpretation.

In response to the reviewer’s comment, we also conducted an additional, more robust analysis using linear mixed models to examine whether expectation level (neutral vs. positive vs. very positive) moderated changes in DASS-21 subscale scores over time, while also controlling for the diagnostic group (depression vs. bipolar disorder). These interaction effects were evaluated separately for the experimental and control groups.

Results indicated that expectation level did not significantly moderate the change in stress, anxiety, or depression scores in either group. However, in the experimental group (five-session condition), we observed non-significant trends suggesting greater symptom reduction among participants with very positive expectations, particularly on the stress and depression subscales. This pattern was not evident in the control group (one-session condition), which could suggest a potential interaction between high expectations and greater intervention intensity, though these findings remain inconclusive.

These moderation analyses and their corresponding estimates are now included in Tables 2 and 5 of the revised manuscript. We believe these additional results enrich the exploratory scope of the study while reinforcing a cautious interpretation of the role of expectations in therapeutic response.

**Reviewer 1 – Comment 7:**
*Several wording issues should also be addressed:*

1. *The term “bilateral” significance level is not standard.*
2. *In the data analysis section, the authors describe a “descriptive analysis” that characterizes the sample, including measures of central tendency and dispersion (percentiles). However, dispersion should be measured by variance/standard deviation when using the mean and by interquartile range when using the median. These essential dispersion measures are missing from the results.*

**Response:**

We thank the reviewer for this helpful comment.

1. We acknowledge that the term “bilateral” significance level is non-standard in English-language scientific writing. In the revised manuscript, we have replaced it with the appropriate term “significance level.”
2. Regarding the descriptive analysis, we agree that the measures of dispersion should align with the type of central tendency used: standard deviation or variance when reporting means, and interquartile range when reporting medians. In the updated version of the manuscript, we have included these essential dispersion measures in the Results section to provide a more complete and accurate summary of the sample characteristics. Specifically, we included means.

These revisions have been implemented to ensure clarity and adherence to statistical reporting standards.

### **Reviewer 2 – Comment 1: Literature review:** *Key literature on music therapy within inpatient settings is missing, as is the unique inpatient context. The authors are encouraged to perform a search on pubmed or similar with terms "music therapy" "mental health" "inpatient" to summarise knowledge to date. See particularly work by Michael J. Silverman (where he has offered many different high intensity interventions), Catherine Carr (see systematic review of music therapy for acute adult psychiatric inpatients in PLOS ONE for an overview of practice and methods up to 2012 -* [*https://pmc.ncbi.nlm.nih.gov/articles/PMC3732280/*](https://pmc.ncbi.nlm.nih.gov/articles/PMC3732280/) *and PhD thesis) and wider considerations of inpatient music therapy practice.*

**Response:**

Thank you very much for this valuable suggestion. We have carefully reviewed the literature on music therapy in inpatient psychiatric settings, particularly the work of Michael J. Silverman and Catherine Carr, as well as relevant systematic reviews. In response to your comment, we have substantially expanded the Introduction section to better contextualize our study within current knowledge and practice.

Specifically, we incorporated the following key references into the revised manuscript:

1. Silverman MJ. Effects of music therapy on psychiatric patients’ proactive coping skills: Two pilot studies. Arts Psychother. 2011;38(2):125-9. doi:10.1016/j.aip.2011.02.004.
2. Silverman MJ. Effects of group songwriting on motivation and readiness for treatment on patients in detoxification: a randomized wait-list effectiveness study. J Music Ther. 2012;49(4):414-29. doi:10.1093/jmt/49.4.414.
3. Silverman MJ. The Influence of Music on the Symptoms of Psychosis: A Meta-Analysis Journal of Music Therapy, XL. 2003. Available from: <http://jmt.oxfordjournals.org/>
4. Carr C, Odell-Miller H, Priebe S. A Systematic Review of Music Therapy Practice and Outcomes with Acute Adult Psychiatric In-Patients. Vol. 8, PLoS ONE. 2013.
5. Gold C, Solli HP, Krüger V, Lie SA. Dose–response relationship in music therapy for people with serious mental disorders: systematic review and meta-analysis. Clin Psychol Rev. 2009;29(3):193-207. doi:10.1016/j.cpr.2009.01.001.

Importantly, our revised introduction now makes clear that this study directly addresses a gap in the literature: the limited evidence regarding the impact of short-term, high-intensity music therapy assited by MIDI during brief psychiatric hospitalizations. We have also included this point in the Discussion section when interpreting our findings.

We are grateful for your guidance, which helped us significantly improve the theoretical framing of our work.

**Reviewer 2 – Comment 2: Procedures:**

*Timing of the outcome assessments is not clear. More generally, the procedure from recruitment into the study, randomisation, intervention delivery and follow-up needs greater clarity. The protocol published under ISRCTN provides an interesting chart suggesting patients were recruited in daily cohorts. Some explanation of how many groups ran (was it one each of high intensity and low intensity or several over the course of the study?) and at what point assessments were taken would be helpful. Please define the time window allowed to collect follow-up data.*

**Response:**

We appreciate this important observation and have revised the manuscript to clarify the procedures regarding recruitment, randomisation, intervention delivery, and follow-up timing.

Patients were consecutively recruited upon admission to the psychiatric unit between August 1st and September 2nd. Inclusion was restricted to those who had been hospitalized for at least 3 to 5 days and showed sufficient clinical stability to participate in up to five music therapy sessions. Upon meeting eligibility criteria, patients were randomly assigned to either the high-frequency (5 sessions/week) or low-frequency (1 session/week) music therapy group.

New intervention cohorts were formed approximately every 4 days, depending on patient admission and availability. The **DASS-21** and **Satisfaction With Life Scale (SWLS)** were administered at two time points:

- **Baseline:** Before the first music therapy session.
- **Post-intervention:** On the fifth day of participation, regardless of group assignment.

The **CISMA scale** was administered in a **pre-post format during the first music therapy session** for both groups. Additionally, for participants in the high-frequency group, the CISMA was also administered in a **second pre-post format during the fifth and final session**.

Follow-up data were collected within a fixed window of **5 days from the start of the intervention**, and patients discharged before completing the post-intervention assessments were excluded from the final analysis.

Importantly, all participants remained in their originally assigned groups throughout the study, and no crossovers occurred. This ensured consistency in exposure to the intervention and the integrity of group comparisons.

These clarifications have been incorporated into the revised manuscript to provide a more complete and transparent description of the study timeline and methodology.

**Reviewer 2 – Comment 3: Procedures:**

*Please provide a brief description of how each of the scales is rated and scored along with psychometric properties.*

**Response:**
We appreciate the suggestion to expand the description of the instruments used in our study. We have included detailed information on the scoring and psychometric properties of each scale into the revised manuscript.

**Reviewer 2 – Comment 4: Procedures:**

*Please provide more detail on your secondary analysis of patient expectations and the methods used to analyse this.*

**Response:**

We appreciate the reviewer’s interest in the secondary analysis of patient expectations. As previously detailed in response to similar feedback from the first reviewer, we have conducted thorough analyses exploring the role of expectations using both correlation methods and more robust linear mixed models that account for intervention group, time, and diagnostic category. These analyses—presented in detail in Tables 5—investigated whether expectation levels moderated changes in clinical outcomes (DASS-21 subscales) over time.

While these exploratory findings suggest possible trends, particularly in the high-intensity intervention group, they do not provide conclusive evidence regarding the direct effect of expectations on treatment efficacy. We have clarified this distinction in the revised manuscript to avoid overinterpretation.

**Reviewer 2 – Comment(s) 4: Intervention:**

*Please use the TiDier template to provide key information about the group music therapy offered in this trial.*

*The intervention described sounds quite unusual in respect to music therapy practice on inpatient wards. To what degree is this approach used in other hospitals? If it is the first time such an approach has been used, this should be made explicit as it limits generalisability. I would also suggest rephrasing of the intervention as group music therapy using MIDI controlled instruments in the title and throughout to make clear that this is one group approach among many.*

*Were multiple participants using a PLAYTRON simultaneously, or was it one controller that was played by one person and others listened? It would help to make clear the extent to which group members were able to jointly create music within sessions.*

*What certification/training does your music therapist hold and how does it relate to professional standards nationally and internationally? This should be stated in the report.*

*Where were the groups held? On or off the ward/ in an open or closed space.*

**Response:**

Thank you for your insightful and constructive comments regarding the description of our intervention. We have carefully addressed each point below to improve clarity and detail, following the TIDieR framework as suggested.

1. **Intervention Description and Use of TIDieR Template**
   We have now incorporated a detailed description of the intervention using the TIDieR template in the revised manuscript, which can be found in an appendix, to clearly outline the rationale, materials, procedures, personnel, delivery mode, setting, duration, and fidelity of the group music therapy using MIDI-controlled instruments. This addition aims to provide a comprehensive and replicable account of the intervention.
2. **Novelty and Generalizability of the Approach**
   We acknowledge that the use of a Playtron MIDI controller connected to conductive objects such as fruits, water, and plants is a novel approach in psychiatric inpatient music therapy, not widely documented in Colombia or internationally. We have explicitly stated in the manuscript that, to the best of our knowledge, this represents one of the first documented uses of such multisensory group music therapy involving MIDI-controlled instruments in a psychiatric inpatient context. We agree this limits generalizability, and we discuss this limitation accordingly.
3. **Terminology and Title Revision**
   In response to your suggestion, we have rephrased the intervention description and title throughout the manuscript to emphasize that this is a *group music therapy using MIDI-controlled instruments*, clarifying that it is one of many possible approaches within music therapy practice. The new suggested title is:

*“****High-Frequency vs. Low-Frequency MIDI-assisted Group Music Therapy in Psychiatric Inpatients: A Randomized Controlled Trial****”*

4. **Group Interaction and Use of PLAYTRON**
   Regarding group participation, multiple patients engaged simultaneously during sessions. The Playtron device was connected to various conductive objects assigned different sounds, allowing ensemble play where up to 16 participants jointly created music in real time. This setup encouraged collaborative creativity, sensory exploration, and group cohesion. We have clarified this interactive and participatory aspect of the intervention in the manuscript.
5. **Music Therapist Certification and Professional Standards**
   The intervention was delivered by a certified music therapist holding a master’s degree in music therapy from the University of La Rioja, Spain, with official validation by the Colombian Ministry of Education. This qualification aligns with national professional standards and international norms within the music therapy community. We have included this certification detail and its relevance to professional standards explicitly in the Methods section.
6. **Location and Setting of Sessions**
   Sessions were conducted in a designated indoor space within the Montserrat Clinic, specifically allocated for occupational therapy with psychiatric inpatients. The setting was a controlled, closed therapeutic environment located on the inpatient ward, designed to ensure safety, privacy, and an appropriate atmosphere for group multisensory music therapy. This clarification has been added to the manuscript to address your query.

We hope these revisions and clarifications satisfactorily address your comments and improve the clarity and rigor of our manuscript.

**Reviewer 2 – Comment (s) 5: Intervention:**

*Given the focus on the difference in intensity it is important to get a sense of how many sessions were available to participants (was it only 5 / 1 session, or could participants attend subsequent weeks ie. if they attended for 2 weeks, they could access up to 10/2 sessions) and the number of sessions actually attended by participants in both arms - please summarise attendance and group size (mean, s.d., range).*

*To what extent did group membership change while the groups were running? A flow of participants would also help to demonstrate the extent group membership was stable or not.*

**Response:**

Thank you for your thoughtful comments regarding the intensity conditions, attendance, and group structure.

We clarify below, incorporating the specific details you requested:

- A total of 91 participants were randomized into either the high-frequency (daily sessions for 5 days) or low-frequency (1 session) intervention groups. Group allocation remained stable, and no crossovers occurred following randomization.
- Three participants (one from each group) withdrew before attending any sessions and were excluded from analysis as shown in the flowchart.
- Among the 89 remaining participants, 74 completed the intervention and post-intervention assessments (n = 37 in each group). Fourteen participants (6 from the high-frequency group and 8 from the low-frequency group) were discharged before post-assessment and therefore excluded from the final analysis.
- Participants in the **high-frequency group** had access to a **maximum of 5 sessions** over 5 consecutive days. Most completed all scheduled sessions unless early discharge occurred. Participants in the **low-frequency group** attended **1 session** during the same 5-day period. After the assessment period, patients from the low-frequency group who remained hospitalized were offered the opportunity to complete additional sessions (up to a total of 5), ensuring equitable access to the intervention; however, these sessions occurred **after data collection** and were **excluded from the analysis**.
- **Group size per session** ranged from 8 to 16 participants, depending on daily availability and discharge timing, with a **mean of approximately 11.4 participants per session** (SD ≈ 3.4).
- Group composition changed modestly during the 5-day period due to routine clinical discharges. Nevertheless, core group membership remained relatively stable across the intervention, particularly in the high-frequency arm, where most participants completed all sessions.

We hope these additions and clarifications fully address your concerns regarding group membership stability and exposure to the intervention.

**Reviewer 2 – Comment 6: Adverse events:**

*It's unusual to have no adverse events in the course of a study. Can you state briefly what sorts of events you assessed for and what might have helped to ensure none occurred during the study.*

**Response:**

Thank you for this important observation. Throughout the study, participants were systematically monitored for potential adverse events. The following categories of events were specifically assessed:

1. Psychological destabilization, including increased anxiety, emotional agitation, exacerbation of mood symptoms, or dissociative reactions during or after sessions.
2. Behavioral disturbances, such as aggression, withdrawal, or refusal to participate.
3. Somatic complaints or physiological symptoms potentially related to overstimulation or discomfort from the multisensory components of the intervention.

Monitoring was performed through direct observation by the session facilitators and through routine communication with nursing staff and attending psychiatrists. Participants were also encouraged to report any discomfort during or after sessions.

No adverse events were observed, reported by participants, or documented by clinical staff during the study period. We believe several factors contributed to this favorable safety profile:

- Participant selection: All individuals were screened by their treating psychiatrists to ensure clinical stability and capacity to participate safely in group therapy.
- Facilitator expertise: Sessions were delivered by a trained music therapist experienced in psychiatric inpatient settings.
- Clinical supervision: A psychiatry resident (principal investigator) was present during all sessions, along with a physician trained in psychological first aid. Nursing staff were also available on-site.
- Structured and supportive environment: Sessions were conducted in a familiar and safe clinical space, with emphasis on gradual engagement and voluntary participation.

Importantly, no participants withdrew from the study due to discomfort or negative emotional reactions, and qualitative feedback suggested a high level of engagement and emotional safety.

**Reviewer 2 – Comment 7: Sample diversity:**

*The discussion would benefit from further detail regarding the sample. Could you explain why numbers of people with psychosis were much lower than depression or anxiety? Similarly it is worth commenting that the study included more women than men. Please also see comment below regarding sampling strategy/length of hospital stay from ISRCTN protocol. It would be helpful to present average length of hospital stay at point of recruitment along with how many were lost during intervention due to discharge.*

**Response:**

Thank you for your helpful comments regarding sample characteristics. The diagnostic distribution in our study reflects the typical clinical profile of patients hospitalized in the psychiatric unit where the study was conducted. Specifically, the predominance of participants with depressive and anxiety disorders is consistent with institutional data showing that affective disorders account for the majority of hospital admissions.

According to internal statistics from the ICSN-Clínica Montserrat, the diagnostic distribution among inpatients is as follows: depressive disorders (58%), anxiety disorders (10%), bipolar affective disorder (13%), and psychotic disorders (8%) **(1)**. This trend is reinforced by the fact that patients with affective disorders tend to have shorter and more stable inpatient courses, making them more likely to be available and clinically eligible for a 5-day intervention. By contrast, patients with primary psychotic disorders are often hospitalized for longer periods, may present with acute symptomatology that limits group participation, and were less frequently available for enrollment during the data collection period.

The overrepresentation of women in our sample (consistent with the 63% female predominance in overall hospital admissions) also reflects the underlying gender distribution of psychiatric inpatients at our institution during the study period **(1)**.

We acknowledge that the use of a convenience sample and a short recruitment window during routine hospitalization limits the representativeness and generalizability of our findings. These aspects are discussed as limitations in the revised manuscript.

**Reference:**

1. Ferro E, Montaña C, González T, Hernández A. Cambios en el perfil epidemiológico, tendencias poblacionales y prestación de servicios de salud mental en el Instituto Colombiano del Sistema Nervioso - Clínica Montserrat entre 2013, 2017 y 2021. PSIMONART. 2022;9(1-2). Disponible en: <https://psimonart.clinicamontserrat.com.co/revista/article/view/5/22>

**Reviewer 2 – Comment 7: Results:**

*There is at least one inaccurate percentage reported in table 1 - suggest these figures are checked.*

*Why are median scores presented in table 2? Mean and s.d. would help to give a better sense of spread of scores.*

*It would be helpful to explain the two assessment points earlier on in the text and how data from each of these points were used.*

**Response:**

We reviewed and corrected Table 1 using R to ensure the accuracy of all percentages and totals. The revised version is now included in the manuscript.

Initially, we reported median scores in Table 2; however, after reassessing the data distribution and incorporating the new data analysis approach suggested by the previous reviewer—as well as in line with your recommendation—we now report means and standard deviations for all scale scores. This change provides a clearer picture of central tendency and variability across groups.

The manuscript has been revised to clearly explain the assessment points for each instrument in previous sections.

**Reviewer 2 – Comment 7: Patient expectancy:**

*Regarding patient expectations of music therapy - did you consider doing a secondary analysis of outcomes, controlling for expectancy rather than a single regression with your overall sample? Do the outcomes change at all?*

**Response:**

Thank you for your insightful follow-up. As detailed in our response to Reviewer 1 (Comment 6), we conducted additional analyses using linear mixed models to assess whether expectation levels moderated outcome changes over time, while controlling for the diagnostic group. These analyses go beyond a single regression and were designed precisely to explore expectancy effects in greater depth. The results and interpretation of these secondary analyses are now reported in table 5 of the revised manuscript, and corresponding clarifications have been added to the Results and Discussion sections.

**Reviewer 2 – Comment 8: Patient expectancy:**

*Lines 312-4 "Additionally, in this study, we found the influence of a suggestive effect of music therapy, since the participants who reported greater expectations before music therapy sessions had better outcomes in depressive symptoms, stress-associated, global life satisfaction, and perception of the impact of music therapy."*

**Response:**

we change all this section to show the new results with the linear mixed models, the secondary analysis of expectations is below table 5.

**Reviewer 2 – Comment 9: Patient expectancy:**

*The association between patient expectations and outcome is hard to disentangle as patients would not have signed up for the study without some positive expectation of attending so the degree to which expectation influences outcome is always going to be limited by a ceiling effect. Both arms have similar proportions of high/medium/neutral expectation so we can say that the differing frequency appears to have an effect but that expectations might inflate this. A different study would be required to test the specific expectancy effects but you could control for expectancy in your results and report whether the effects you initially report hold true (but with the limitations that this is a secondary/post-hoc analysis).*

**Response:**

We appreciate this thoughtful observation regarding the complex role of patient expectations. Indeed, as noted, all participants voluntarily agreed to participate in the study, which implies a baseline level of positive expectation that may limit variability and introduce potential ceiling effects.

To address this, we conducted additional secondary analyses using linear mixed models that included expectation level (neutral, positive, very positive) as a covariate, while also controlling for the diagnostic group. These analyses did not reveal a statistically significant moderating effect of expectations on clinical outcomes in either intervention arm. However, we did observe non-significant trends in the high-frequency group suggesting that participants with very positive expectations may have experienced greater symptom reductions in stress and depression scores. These findings, while inconclusive, highlight the potential interaction between treatment intensity and patient attitudes, and are now reported in Tables 5.

We agree that a study specifically designed to isolate expectancy effects would be required to draw definitive conclusions. In the current study, the relatively balanced distribution of expectation levels between groups helps reduce the likelihood that differences in outcomes are solely attributable to expectancy. Nonetheless, we acknowledge that expectations may have contributed to the observed changes and have clarified this limitation in the discussion.

**Reviewer 2 – Comment 10: Patient expectancy:**

*Lines 279-280:"Positive correlations were found, employing a matrix, between the expectation of music therapy and final life satisfaction (P<0.001), CISMA final total score in the first (P=0.005) and last measurement (P=0.001)" - I did not understand this. Please explain/make it clearer.*

**Response:**

We appreciate the opportunity to clarify this section. The original sentence referencing a "matrix" of correlations was ambiguous and has now been revised for clarity. In response to your comment—and in line with Reviewer 1’s prior suggestion—we conducted a more robust analysis using linear mixed models to explore the relationship between expectation levels and perceived change, as measured by selected CISMA items (specifically items 1, 3, and 4). These models included time as a covariate and were used to assess within-group changes and potential group differences.

Although the residuals did not meet strict normality assumptions, the diagnostic plots supported the model fit, and the use of mixed models allowed us to better account for repeated measures and individual variability. The results indicated no statistically significant differences between groups post-intervention on CISMA items, although small non-significant trends favored the high-frequency group. These updated findings are now described in the Results section and provide a more cautious and accurate interpretation of the relationship between expectations and perceived therapeutic effects.

We have also removed the confusing reference to a “matrix of correlations” and have revised the manuscript to more precisely reflect the analytical approach taken.

**Reviewer 2 – Comment 11: Discussion:**

*As per literature review/introduction, this needs re-writing with a stronger focus on comparison with existing music therapy/mental health papers rather than wider studies.*

**Response:**

Thank you for the observation. In response, we have revised the discussion section to better align with the existing literature on music therapy in mental health contexts. Specifically, we incorporated studies that evaluated the effects of music therapy in psychiatric populations, including individuals with depression and post-traumatic stress disorder. These additions strengthen the comparison between our findings and prior research, highlighting the relevance and consistency of our results within the field of music therapy for mental health.

Additionally, we acknowledge that the relatively brief and moderate-intensity nature of our intervention may explain the lack of significant effects on more stable outcomes such as global life satisfaction. We suggest future studies could assess whether music therapy may contribute to reduced pharmacologic needs—particularly in short-acting anxiolytic use—over longer periods.

We believe these enhancements to the Discussion offer a more rigorous and clinically grounded interpretation of our findings in the context of the existing music therapy literature, as requested.

**Reviewer 2 – Comment 12: Discussion:**

*Line 316-7 "When evaluating the intensity of sessions needed to find an effect, the meta-analysis of Aalbers et al. found that adding twelve music therapy sessions to the usual treatment is more effective in reducing symptoms of depression, anxiety, and improved functioning (21)" - I could not find this information in the referred paper. Please check the citation. You might also want to refer to Gold et al's paper on dose response:* [*https://pubmed.ncbi.nlm.nih.gov/19269725/*](https://pubmed.ncbi.nlm.nih.gov/19269725/)

**Response:**

Thank you very much for your careful reading and valuable observation. You are correct — the specific statement referring to “twelve sessions” and their effect was inaccurately attributed to the meta-analysis by Aalbers et al. (2017). After reviewing the cited source, we confirm that it does not provide a specific analysis or recommendation regarding the number or intensity of music therapy sessions required to observe significant effects. We have therefore removed the reference to the number of sessions from the manuscript to avoid any misrepresentation. The revised sentence now more accurately reflects the content of the cited meta-analysis:

"This aligns with the findings of the meta-analysis by Aalbers et al. (2017), which concluded that adding music therapy to treatment as usual is more effective than standard treatment alone in reducing depressive symptoms and improving functioning.."

Also, as mentioned above, we took into account the ***Gold et al.*** study for the restructuring of the introduction and discussion in the revised manuscript. We appreciate your feedback, which helped us correct this and improve the accuracy of the manuscript.

**Reviewer 2 – Comment 13: Comparison with published protocol on ISRCTN:**

*"All ppt will participate in all intervention modes high and low intensity" – did high intensity also have low intensity offered afterwards or did your protocol mean just that low intensity participants were offered high intensity afterwards? How did this fit with assessments, and did this mean that additional participants (not being assessed) were in later groups?*

**Response:**

Thank you for your close reading of the protocol. We clarify that only participants initially randomized to the low-intensity group (one session) were offered the opportunity to complete additional sessions during the following week, but only if they remained hospitalized and expressed interest. This option allowed them to receive the full set of 5 sessions, mirroring the high-intensity group, but it occurred after the conclusion of outcome assessments.

Importantly, these additional sessions were not part of the original randomization protocol and were conducted after the Day 5 assessments (DASS-21 and SWLS) had been completed. Therefore, they were not included in any efficacy analyses. Participants in the high-intensity group were not offered any further sessions beyond their original five-session intervention.

No additional participants were enrolled beyond the originally randomized sample described in the flowchart, and this optional extension did not affect data collection or study outcomes.

**Reviewer 2 – Comment 14: Comparison with published protocol on ISRCTN:**

*"Priority will initially be given to patients with longer hospitalization durations to maximize intervention exposure. As recruitment progresses and patients with extended stays are enrolled, focus will gradually shift toward those with shorter hospitalizations. This approach is designed to maintain consistent group sizes for the intervention sessions, facilitating robust and enriched group therapy sessions throughout the recruitment period."

Please ensure this detail is in the procedures section of the paper - and also acknowledged as a limitation.*

**Response:**

Thank you for the observation. We have clarified this point in the Procedures section, noting that recruitment focused on patients hospitalized between days 3 and 5 to ensure intervention completion. Additionally, we recognize limitations related to the sensitivity of the measurement instruments (SWLS, CISMA) and the lack of systematic pharmacological data, which may have influenced clinical outcomes.

___________________________________________________________________

We sincerely thank the reviewers and editors for their thoughtful and constructive comments, which have greatly contributed to improving the quality and clarity of our manuscript. We have carefully addressed each point and revised the manuscript accordingly. We believe these changes strengthen the study and its presentation. We look forward to your further feedback and hope the revised manuscript meets the standards for publication.

Thank you for your time and consideration.

Sincerely,

**Eugenio Ferro, MD.**
