# Supplementary material for: High-frequency vs. low-frequency MIDI-assisted group music therapy in psychiatric inpatients: A randomized controlled trial
Source: PLoS One. 2026 Apr 17;21(4):e0317950. doi: 10.1371/journal.pone.0317950 (PMC13089878; doi:10.1371/journal.pone.0317950)
Supplement: Attachment — Submitted filename: Rebuttal Letter_13Jan2026.docx [file pone.0317950.s007.docx]

**RE: Manuscript Title**: "**High-Frequency vs. Low-Frequency MIDI-assisted Group Music Therapy in Psychiatric Inpatients: A Randomized Controlled Trial**"

We would like to thank the Academic Editor and the reviewer for their careful evaluation of our manuscript entitled *“High-Frequency vs. Low-Frequency MIDI-assisted Group Music Therapy in Psychiatric Inpatients: A Randomized Controlled Trial”* (Manuscript ID: PONE-D-24-55131R1).

We are grateful for the detailed and constructive comments, which have been invaluable in improving the clarity, methodological rigor, and overall quality of the manuscript. We have revised the manuscript extensively in response to all points raised.

Below, we provide a point by point response to each comment from the Academic Editor and the reviewer. A marked-up version of the revised manuscript with track changes enabled has been submitted as “Revised Manuscript with Track Changes”, allowing all modifications to be easily identified. In addition, a clean version of the revised manuscript, without tracked changes, has been submitted as “Manuscript”.

**Reviewer 1 – Comment 1:**

The abstract is not appropriately written. Please use the CONSORT extension for abstracts. Important information is missing, including details on allocation concealment and the timing of the intervention. One sentence (line 36/37) is still written in the future tense and should be revised. The results section of the abstract is too brief; numerical results rather than only p-values should be reported.

Furthermore, the abstract should not present figures describing changes over time within groups, but should instead present between-group differences to make clear that—with one exception—no differences between the groups were found across all outcomes. This also needs to be reflected in the abstract’s discussion. At present the abstract appears to suggest positive effects, but as this is a randomized controlled trial and not a before-after–post study, this distinction must be made explicit. As in other parts of the manuscript, the DASS instrument is incorrectly abbreviated as “DAS” here as well.

**Response:**

We thank the reviewer for this detailed and constructive feedback. The abstract has been thoroughly revised in accordance with the CONSORT extension for abstracts. Specifically, we have clarified the randomized controlled trial design, explicitly described allocation concealment and the timing of the intervention, and corrected the abbreviation of the Depression Anxiety Stress Scale to DASS-21 throughout the manuscript.

The Results section of the abstract has been expanded to report numerical between group estimates rather than p-values alone, and the focus has been shifted from within group changes to between group differences, as appropriate for a randomized controlled trial. The wording of the Conclusions has also been revised to avoid a before and after interpretation and to clearly state that, with the exception of stress symptoms, no significant between-group differences were observed across outcomes. All future-tense phrasing has been corrected.

We believe these revisions substantially improve the clarity, methodological accuracy, and CONSORT compliance of the abstract.

**Reviewer 1 – Comment 2:**

The manuscript should state and explain why the trial registration was conducted post hoc. This is a major limitation that should be clearly emphasized. The manuscript also refers to a study protocol; however, registration is not a study protocol. It appears that no protocol exists, which must be stated.

**Response:**

We thank the reviewer for these related comments and agree that clearer distinctions were needed between trial registration and the existence of a study protocol. We have revised the manuscript to explicitly state that the trial was registered retrospectively, which constitutes a major methodological limitation, and that no publicly registered protocol exists. We now clarify that trial registration and protocol development are distinct processes, and that retrospective registration does not replace a prospectively registered protocol.

We further explain that, within the Colombian regulatory context, prospective registration of clinical trials on international platforms is not a mandatory requirement. However, we emphasize that this regulatory context does not mitigate the methodological limitation associated with retrospective registration, which restricts public verification of the study timeline and pre-specified methods.

At the same time, we clarify that the study was conducted under a complete written internal protocol developed prior to data collection, which specified the study design, intervention procedures, outcomes, and analysis plan, and which was reviewed and approved by an independent Ethics Committee in accordance with national regulations. Nevertheless, we now explicitly acknowledge that this protocol was not publicly accessible.

These revisions were made to improve conceptual clarity and transparency and to align the manuscript with CONSORT recommendations.

**Reviewer 1 – Comment 3:**

Please revise the manuscript in accordance with the current CONSORT 2025 Statement, which contains elements that have not yet been considered. In the submitted CONSORT checklist, “not applicable” is listed under blinding. This is inappropriate. The lack of blinding should be described in the Methods section, not only in the Discussion.

**Response:**

We thank the reviewer for this important comment. The manuscript has been revised in accordance with the CONSORT 2025 Statement, and the reporting of methodological details has been updated accordingly.

Specifically, the issue of blinding has now been explicitly addressed in the Methods section. A dedicated subsection (“Blinding”) was added to clarify that blinding of participants and therapists was not feasible due to the nature of the music therapy intervention. We now clearly describe that participants and the music therapist were aware of group allocation, while participant recruitment and enrollment were conducted by a coordinator blinded to the randomization sequence, which was generated independently and not accessible during enrollment. We also clarify that outcomes were assessed using self-reported questionnaires and that no formal blinding of outcome assessment was implemented.

In addition, the CONSORT checklist has been corrected to remove “not applicable” under blinding and now accurately reflects the procedures described in the Methods section. These revisions were made to improve transparency and ensure full compliance with CONSORT 2025 reporting standards.

**Reviewer 1 – Comment 4:**

The background section cites several older reviews and original studies. This should be revised to include more recent work, particularly the reviews by Aalbers and Zhong, which are already cited in the discussion. The section added in the revision describing Silverman’s studies (from line 116 onward) should be removed, as these studies were already included in the review by Carr mentioned before. The reference to Goldberg’s meta-analysis should also be removed. Although it is described as “recent evidence,” it was published in 2009 and is therefore outdated.

**Response:**

We have removed the older reviews and original studies identified by the reviewer from the Background section. Specifically, the section describing Silverman’s individual studies has been deleted, as these studies were already synthesized within previously cited reviews, and the reference to Goldberg’s meta-analysis (2009) has also been removed.

The Discussion has been updated to reflect more recent evidence, with citations primarily from 2020 onward, which now frame the interpretation of the findings.

**Reviewer 1 – Comment 5:**

The final paragraph of the introduction (from line 134 onward) should be removed.

**Response:**

The final paragraph of the Introduction (from line 134 onward) has been removed in the revised manuscript, as requested.

**Reviewer 1 – Comment 6:**

The Methods section is generally complete, aside from the missing information on blinding (see above). However, it requires more structure, and the CONSORT 2025 Statement can serve as a helpful guide for organizing the content and reducing redundancies, which currently occur frequently, for example, in the descriptions of randomization and allocation concealment, which are presented in varying detail across different sections.

**Response:**

The Methods section has been restructured in accordance with the CONSORT 2025 Statement. Redundant descriptions of randomization and allocation concealment have been removed and consolidated into a single dedicated subsection. Information on blinding has been clarified and organized following CONSORT recommendations. Overall, the section was reorganized to improve clarity, reduce repetition, and enhance transparency.

**Reviewer 1 – Comment 7:**

The sample size calculation should be explicitly labelled as such, rather than described merely as “sample.”

**Response:**

Thank you for this comment. We have clarified the manuscript by explicitly labelling the subsection as “Sample size calculation”, in accordance with CONSORT recommendations. In addition, we clarified in the Participants section that the description of the inpatient population refers exclusively to the study setting and eligibility context, and not to the sample size estimation procedure. This distinction was made to avoid ambiguity between the a priori statistical calculation and the characteristics of the enrolled participants.

**Reviewer 1 – Comment 8:**

The text beginning at line 167 does not belong in the Methods section, as it describes characteristics of the sample.

**Response:**

The text beginning at line 167 has been removed from the Methods section and relocated to the Results section, where sample characteristics are reported.

**Reviewer 1 – Comment 9:**

In line 196, the manuscript refers to “8 periods over 6 weeks.” However, the study was conducted only from 1 August to 2 September, so it is unclear how 6 weeks of data collection were possible.

**Response:**

The wording in line 196 has been revised to avoid ambiguity. The text “8 periods over 6 weeks” referred to overlapping time periods of group cohorts recruited. The reference to “six weeks” has been removed, and the text now clarifies that the eight periods correspond to sequential recruitment blocks formed approximately every four days during the enrollment phase (1 August to 2 September), rather than to follow-up or observation periods.

**Reviewer 1 – Comment 10:**

As this is a complex intervention, the authors should consider presenting the intervention in accordance with the TIDieR checklist. If appropriate, the description of the intervention from line 313 onward could be expanded based on TIDieR.

**Response:**

We made a supplementary material that provides a structured and detailed description of the intervention components, procedures, materials, delivery, and dosage, following the TIDieR framework. However, it is too long to inclue it in the manuscript text. In the manuscript, we described the intervention in accordance with the TIDieR checklist, adapted to make it easy for the readers. We have now explicitly referenced this appendix in the Methods section to improve clarity and accessibility for readers (page 13 of the final manuscript).

**Reviewer 1 – Comment 11:**

The predefined primary endpoint is the DASS scale, which consists of three components. However, these three components were analyzed separately, and only one of them showed significant differences. The authors should consider whether this constitutes multiple testing and whether a Bonferroni correction should be applied to the primary endpoint. With three primary endpoints, the adjusted p-value threshold would be 0.0125 instead of 0.05. At minimum, this issue should be critically reflected upon and discussed.

**Response:**

We agree that the analysis of the three DASS-21 subscales raises the issue of multiplicity if they are interpreted as independent primary endpoints.

In this trial, the DASS-21 was defined as a multidimensional primary outcome, reflecting a single overarching construct of psychological distress operationalized through three conceptually and statistically interrelated subscales (depression, anxiety, and stress). Accordingly, the subscales were treated as correlated coprimary endpoints, which is common practice in clinical trials using multidimensional psychometric instruments.

Bonferroni correction assumes statistical independence among tests and is known to be overly conservative when applied to correlated measures derived from the same instrument. Given the moderate-to-high intercorrelations among DASS-21 subscales consistently reported in the literature, applying a Bonferroni-adjusted threshold would substantially increase the risk of Type II error and potentially obscure clinically meaningful effects. In addition, the use of linear mixed-effects models partially accounts for shared variance across repeated measures.

Nonetheless, we acknowledge that the analysis of multiple subscales entails a risk of multiplicity. In response to the reviewer’s comment, we have explicitly addressed this issue in the Discussion, emphasizing that the between-group interaction observed for the stress subscale should be interpreted cautiously and as suggestive rather than definitive evidence, particularly in light of multiple testing and other methodological limitations.

**Reviewer 1 – Comment 12:**

The effects need to be discussed much more cautiously given the numerous limitations. For example, line 560 suggests “a clear effect,” whereas the results more appropriately indicate “suggestive” evidence rather than clear effects.

**Response:**

We agree with the reviewer that the interpretation of the findings required a more cautious tone given the study’s methodological limitations. In response, we have revised the Discussion section to remove language implying clear or definitive effects, including the statement previously referring to a “clear effect.” The findings are now consistently described as suggestive and exploratory, and the conclusions have been aligned with the acknowledged limitations of the study. These revisions ensure that the interpretation is proportional to the strength of the evidence and consistent with CONSORT recommendations.

**Reviewer 1 – Comment 13:**

The newly added discussion of the studies by Carr and Silverman (from line 644 onward) should be removed. I acknowledge that this was added in response to a previous review, but these studies are relatively old, and newer evidence is available; therefore, this section should be deleted.

**Response:**

In response, we have removed the discussion of the studies by Carr and Silverman from the manuscript, as recommended. This section has been deleted in its entirety.

To ensure that the Discussion remains grounded in the most current evidence, we have revised the relevant paragraphs and incorporated more recent systematic reviews, meta-analyses, and controlled studies addressing the effects of music therapy in acute psychiatric settings and the role of intervention intensity. These updates allow the Discussion to reflect contemporary evidence while maintaining coherence with the study findings.

**Reviewer 1 – Comment 14:**

A very important limitation that must be added is that participants who did not receive the intervention were excluded from the analysis. This means that the study did not conduct an intention-to-treat analysis but a per-protocol analysis. This is a major limitation that should be prominently highlighted.

**Response:**

We agree that the analytic approach corresponds to a per-protocol framework, because participants who did not receive any intervention were excluded from the final analysis. However, this decision is directly aligned with the original design and scientific objective of the study, which from the title, aim, and protocol stage focused specifically on comparing two active intervention conditions: high-frequency versus low-frequency music therapy.

Participants who did not attend any session could not be meaningfully classified into either intensity group, and retaining them in an intention-to-treat analysis would have introduced bias in estimating the dose–response relationship inherent to the primary research question. Nonetheless, we fully acknowledge that analyzing only participants who received the allocated intervention reduces generalizability and may introduce selection bias. We have therefore added this as an explicit limitation in the manuscript, as suggested.

**Reviewer 1 – Comment 15:**

Although the lack of blinding is mentioned as a limitation, the authors should also discuss whether it would have been possible to include additional, potentially more objective outcomes, such as length of stay, effectiveness of psychiatric treatment, or medication use. These could at least have been included as secondary outcomes to reduce the risk of bias associated with the lack of blinding.

**Response:**

We agree that the inclusion of additional objective outcomes, such as length of hospital stay, psychiatric treatment response, or changes in medication use, could have strengthened the study by reducing the risk of bias inherent to the lack of blinding. However, the present trial was designed specifically to evaluate the short-term psychological impact of music therapy sessions, and its scope did not originally include these broader clinical indicators.

Nonetheless, we fully recognize the importance and relevance of such objective measures, and we have now added a statement in the Discussion highlighting that future studies should incorporate these outcomes as secondary endpoints. Doing so would improve methodological robustness and provide a more comprehensive assessment of the therapeutic benefits of music therapy in psychiatric inpatients.

We have revised the manuscript accordingly (see Discussion section).

**Reviewer 2 – Comment 1:**

The authors report a sample size of n = 30 per group based on the specifications (α = 0.05, power = 0.8, Cohen’s *d* = 0.6, group ratio = 1:1). However, my calculations using both G*Power and R’s pwr package (function: pwr.t.test()) yield different estimates:
• Two-tailed test: n = 45 per group.
• One-tailed test: n = 36 per group.
Could the authors check their sample size estimate and also clarify whether a one- or two-tailed test was assumed?

**Response:**

We appreciate the reviewer’s careful verification of the sample size calculation. We re-evaluated our assumptions using R’s pwr.t.test() function. For a two-tailed test with α = 0.05, 80% power, and Cohen’s d = 0.6, the required sample size is indeed approximately 45 participants per group.

However, our original conceptual hypothesis was directional: we expected that the high-frequency music therapy group would show a greater reduction in psychological distress compared with the low-frequency group, based on consistent evidence from prior clinical and psychotherapeutic music interventions. Given the absence of theoretical or clinical rationale for a detrimental effect of higher session frequency, a one-tailed framework is justifiable. Under a one-tailed assumption, the required sample size is approximately 36 participants per group, which matches closely the 37 per group included in the study.

To avoid ambiguity, we have now clarified in the Methods section that a directional hypothesis underlies the interpretation of the primary endpoint, and we revised the sample size calculation accordingly (see Methods → Sample size calculation).

**Reviewer 2 – Comment 2:**

I raised the issue of not using repeated measures analysis in my previous round of comments. I appreciate the authors taking my advise and revised their entire analysis. However, this change introduces an inconsistency between the sample size justification and the actual analytical model – the sample size is justified based on a simple independent sample t-test while the analysis is a much more complicated linear mixed model. I understand that power analysis for linear mixed model is more complex. I recommend that the authors to acknowledge this discrepancy and comment briefly on potential power limitation. A post hoc power analysis may also be considered.

**Response:**

We appreciate the reviewer’s insightful observation. We agree that there is a methodological discrepancy between the a priori sample size estimation—which was based on a simple two-sample t test—and the final analytical approach, which required a repeated-measures structure using linear mixed models (LMM). At the time of study planning, analytical decisions were based on the assumption of a pre–post comparison of independent groups, while power analysis for LMM is considerably more complex and was not feasible with the resources available during protocol development.

We have now explicitly acknowledged this issue in the manuscript and clarified that the use of LMM may influence the effective statistical power. Given the achieved sample size, the study may have been underpowered to detect medium effects in the interaction term. We added a short statement in the Limitations section addressing this issue.

Regarding post hoc power analysis, we note that such analyses are generally discouraged as they do not provide additional insight beyond the observed effect sizes and confidence intervals. However, we agree on the importance of transparency and have added a brief statement contextualizing the achieved sample size relative to the expected effect size (seeMethods).

**Reviewer 2 – Comment 3:**

Additionally, the authors stated that the model diagnostics supported the use of linear mixed model due to homoscedastic residuals and the absence of influential outlier. Can the authors provide supports of these statements in the supplementary documents?

**Response:**

We thank the reviewer for this important comment. In response, we have conducted a comprehensive re-evaluation of the diagnostic assumptions for all linear mixed-effects models used in the study.

Detailed graphical and numerical diagnostics have now been added as **Supplementary Material S2**. This supplementary file includes QQ-plots of standardized residuals and residuals-versus-fitted plots for each outcome (depression, anxiety, and stress) and intervention group, as well as a summary table of standardized residual diagnostics.

Across all models, residuals showed an approximately normal distribution with no extreme standardized residuals (|z| > 3), and residuals-versus-fitted plots demonstrated random and symmetric dispersion around zero, supporting the assumption of homoscedasticity. In addition, influence diagnostics indicated the absence of influential observations.

These supplementary materials provide explicit empirical support for the appropriateness and robustness of the linear mixed-effects modeling approach used in this study.

**Reviewer 2 – Comment 4:**

Only three items from the CISMA instrument were used. Why were only these three items selected? Additionally, is was stated that the CISMA has shown strong content validity and responsiveness in clinical music therapy practice. Any supports for this statement?

**Response:**

We have clarified the rationale for selecting three CISMA items in the Methods section, emphasizing their relevance for capturing immediate, session-specific effects of music therapy and minimizing participant burden. Additionally, we have revised the manuscript to provide appropriate support for the CISMA’s content validity and responsiveness, citing prior clinical and mixed-methods studies, while acknowledging the need for further psychometric validation.

**Reviewer 2 – Comment 5:**

A single item was used for measuring overall SWLS. It was stated that “factor analysis also supports its reliability with communalities of 0.70 and 0.77”. How was the factor analysis conducted with only one item? Also, what does “corrected attenuation” mean? It is also strange to argue for construct validity for a single item. These statements suggest that the authors may have some misconceptions of psychometric properties.

**Response:**

Thank you for this clarification. We agree that psychometric properties such as factor analysis, communalities, corrected attenuation, and construct validity apply to multi-item instruments rather than to a single-item measure used in isolation. While Reference 25 discusses these properties in the context of scale development and validation, we acknowledge that our original wording may have inaccurately extended these concepts to the single-item measure used in our study.

We have revised the manuscript to remove these statements and to clarify that the single SWLS item was used solely as a brief, exploratory indicator of global life satisfaction, consistent with methodological recommendations for minimizing respondent burden in clinical settings. This limitation is now explicitly acknowledged in the Discussion.

**Reviewer 2 – Comment 6:**

The authors did not adequately address the issue of multiple comparison bias raised in my previous comments. While a linear mixed model was used, this does not inherently correct for the increased risk of false positives when testing multiple outcome variables. If a Bonferroni correction (adjusting for four comparisons) were applied, the significance threshold would be reduced to p < 0.0125 (0.05/4), rendering the reported findings non-significant. This underscores the need for caution in interpreting the results, as they may be susceptible to Type I error inflation.

**Response:**

We appreciate the reviewer’s continued attention to the issue of multiplicity. We agree that testing several outcomes increases the risk of Type I error and that this must be acknowledged transparently.

In the present study, the three DASS-21 subscales stem from a single psychometric instrument and represent correlated dimensions of the overarching construct of psychological distress, rather than independent endpoints. Because Bonferroni correction assumes statistical independence across tests, its application in this context is known to be overly conservative and may increase the risk of Type II error, potentially masking clinically meaningful effects.

Nonetheless, we acknowledge that the interaction effect observed in the stress subscale (p = 0.023) does not meet a Bonferroni-adjusted threshold, and we now explicitly state this limitation in the Discussion to encourage cautious interpretation. Importantly, all three DASS-21 domains demonstrated significant within-group improvements, supporting the overall therapeutic effect of music therapy, although only the stress domain showed a between-group differential effect.

In response to the reviewer’s request, we have revised the manuscript to (i) explain why Bonferroni correction may not be the optimal method for correlated psychometric outcomes, and (ii) clarify that the stress-domain interaction should be interpreted conservatively given the multiplicity of tests. These additions appear in the Discussion section.

___________________________________________________________________

We have revised the manuscript in response to the reviewers’ comments and have addressed each point accordingly. The revisions have focused on improving clarity, methodological transparency, and alignment with reporting standards. We believe these changes have strengthened the manuscript and its presentation.

Thank you for your time and consideration.

Sincerely,

Eugenio Ferro, MD.
