# Supplementary material for: High-frequency vs. low-frequency MIDI-assisted group music therapy in psychiatric inpatients: A randomized controlled trial
Source: PLoS One. 2026 Apr 17;21(4):e0317950. doi: 10.1371/journal.pone.0317950 (PMC13089878; doi:10.1371/journal.pone.0317950)
Supplement: Attachment — Submitted filename: Rebuttal Letter_18Feb2026.docx [file pone.0317950.s008.docx]

**RE: Manuscript Title**: "**High-Frequency vs. Low-Frequency MIDI-assisted Group Music Therapy in Psychiatric Inpatients: A Randomized Controlled Trial**"

We would like to thank the Academic Editor for your careful evaluation of our manuscript submitted to PLOS ONE, entitled *“High-Frequency vs. Low-Frequency MIDI-assisted Group Music Therapy in Psychiatric Inpatients: A Randomized Controlled Trial”* (Manuscript ID: PONE-D-24-55131R1).

Below, we respond to the comment from the Academic Editor. A marked-up version of the revised manuscript with track changes enabled has been submitted as “Revised Manuscript with Track Changes”, allowing all modifications to be easily identified. In addition, a clean version of the revised manuscript, without tracked changes, has been submitted as “Manuscript”.

**Academic editor – Comment 1:**

The information we provided in the ‘Funding Information’ and ‘Financial Disclosure’ sections does not match.

**Response:**

We revised the information we provided in the ‘Funding Information’ and ‘Financial Disclosure’.

We want to highlight that this study did not receive any external grant funding or any financial support from any institution. We recognized the health institution (Clinica Montserrat – Hospital Universitario) with an acknowledgement for their support and facilities, but we did not receive any financial support.

We mentioned Instituto Colombiano del Sistema Nervioso, Clinica Montserrat, in the submission platform, because the institution approved the conduction of the trial in their facilities, under Acta 189 18-JUN-2024, but did not give any financial support. The authors covered all project costs. Only the authors had a role in the study design, data collection and analysis, decision to publish, and preparation of the manuscript.

Also, we included in the cover letter the following “Funding disclosure statement”:

“This study did not receive any external grant funding. The authors covered all project costs. No institution had any role in the study design, data collection and analysis, decision to publish, or preparation of the manuscript. The authors declare that they have no financial or non-financial competing interests.”

Thank you for your time and consideration.

Sincerely,

Eugenio Ferro, MD.

Corresponding author

eferro@unbosque.edu.co
